# A versatile bioelectronic interface programmed for hormone sensing

**DOI:** 10.1038/s41467-023-39015-1

**Published:** 2023-05-31

**Authors:** Preetam Guha Ray, Debasis Maity, Jinbo Huang, Henryk Zulewski, Martin Fussenegger

**Affiliations:** 1 ETH Zurich, Department of Biosystems Science and Engineering, Mattenstrasse 26, CH-4058 Basel, Switzerland; 2 Division of Endocrinology, Diabetes and Metabolism, University Hospital Basel, Petersgraben 4, CH–4031 Basel, Switzerland; 3 Division of Endocrinology and Diabetes, Stadtspital Triemli, Birmensdorferstrasse 497, CH–8063 Zurich, Switzerland; 4 Faculty of Science, University of Basel, Mattenstrasse 26, CH-4058 Basel, Switzerland

**Keywords:** Synthetic biology, Type 1 diabetes, Sensors and probes

## Abstract

Precision medicine requires smart, ultrasensitive, real-time profiling of bio-analytes using interconnected miniaturized devices to achieve individually optimized healthcare. Here, we report a versatile bioelectronic interface (VIBE) that senses signaling-cascade-guided receptor-ligand interactions via an electronic interface. We show that VIBE offers a low detection limit down to sub-nanomolar range characterised by an output current that decreases significantly, leading to precise profiling of these peptide hormones throughout the physiologically relevant concentration ranges. In a proof-of-concept application, we demonstrate that the VIBE platform differentiates insulin and GLP-1 levels in serum samples of wild-type mice from type-1 and type-2 diabetic mice. Evaluation of human serum samples shows that the bioelectronic device can differentiate between samples from different individuals and report differences in their metabolic states. As the target analyte can be changed simply by introducing engineered cells overexpressing the appropriate receptor, the VIBE interface has many potential applications for point-of-care diagnostics and personalized medicine via the internet of things.

## Abstract (web summary)

Ultrasensitive, real-time profiling of bio-analytes is a prerequisite for precision medicine. Here, the authors present a versatile bio-electronic interface (VIBE) to sense signaling cascade-guided receptor-ligand interactions and show that it can detect hormone levels in blood samples and differentiate individual metabolic conditions.

## Introduction

Precision-guided personalized long-term treatment of chronic disorders will require systems that can accurately sense a target analyte and regulate the delivery of a therapeutic protein. However, to optimize the dosage and timing, we require a versatile electronic platform sensitive to changing concentrations of small molecules, such as hormones, to serve as an interface between the electronic and genetic components. Here, we aimed to meet this need by developing a versatile bioelectronic interface (VIBE) platform with segregated components capable of sensing engineered signaling-cascade-guided receptor–ligand interactions via an electronic interface. We focused initially on diabetes as a serious and common disease caused by dysregulation of glucose metabolism. Insulin and GLP-1 are primary regulators of glucose metabolism^1–3^, and abnormal levels of insulin or GLP-1 are also associated with cardiovascular or neuronal disorders^4–7^. Though analytical methods such as enzyme-linked immunosorbent assay (ELISA) and radio-immunoassay provide high selectivity for insulin and GLP-1, electronic interfaces have the potential to provide compact, ultra-sensitive, real-time detection and monitoring systems, such as personalized medical devices^8,9^.

In pioneering work, Cox and Gray developed ruthenium-based films for direct electrochemical detection of insulin at acidic and physiological pH, and these films were subsequently further improved^10–12^. Wang et al. introduced a method based on direct catalytic oxidation of insulin using carbon nanotubes (CNTs)^13,14^, and later CNTs modified with chitosan or nickel-cobalt oxide composite with a Nafion coating were used for direct oxidation of tyrosine residues of insulin^15,16^. Similarly, Rafiee et al. developed a nickel oxide nanoparticle-modified multiwalled carbon nanotube-Nafion composite coated onto screen-printed electrodes and Snider et al. reported nanotube-dihydropyran composite electrode-guided detection of insulin in a microfluidic chip^17,18^. However, these techniques involve direct oxidation of amino acid residues (primarily tyrosine) of insulin, and it is a major challenge to avoid the oxidation of amino acid residues of other serum proteins as well; indeed, application of these methods has so far been limited to buffered solutions.

Subsequently, nanotube-based electrodes modified with monoclonal antibodies or electrodes modified with free-standing aptamers were used to capture insulin on the electrode surface, and the resulting suppression of signal output was measured for real-time detection^19–21^. Although the results were promising, such surface modification of electrodes or nanotubes often leads to steric hindrance, conformational changes, or a poor ability to withstand incidental physical forces during experiments. A silicon-based field-effect transistor with multiple channels was also developed to detect insulin in the sub-femtomolar range^22^, but high dilution (x10,000) is required to reduce noise and prevent clogging. Optical approaches using surface plasmon resonance (SPR) have also been reported^23^, and hetero-bifunctional gold (Au) films were introduced to increase the detection efficiency^24^. For next-generation SPR-based indirect immunoassays, Au-nanoparticle-based bifunctional dendrimers were self-assembled into monolayers to achieve higher stability and binding efficiency and minimize non-specific interaction^25^. Magnetic nanoparticle-based direct and sandwiched assays were also explored^26^. However, instability, misfolding, and reversed orientation of the capture molecules on SPR surfaces decrease the quality, sensitivity, and specificity of the signal readout and represent major challenges in the manufacturing and clinical validation of state-of-the-art sensor devices tailored for the quantification of relatively small molecules such as peptide hormones^27^. Dissecting surface electronics from the biological sample and measuring the ligands in a native context of human cells may improve precision, specificity, and robustness while increasing the multiplexing and simplicity of the overall assay format. We considered that this approach, using engineered cell lines expressing specific receptors as capture probes, might be applicable to the construction of interconnected miniaturized devices that could have many potential applications in point-of-care diagnostics and personalized medicine for smart, ultrasensitive, and real-time screening of bio-analytes.

In this context, engineering of the cells with specific receptors rewired to natural and synthetic trans-activators or signaling cascades for transgene expression is quite well established^28,29^. For example, Ye et al. described a self-adjustable genetic circuit with insulin receptors and a synthetic transactivator domain for autonomous control of transgene expression to correct insulin resistance^30^. Xie et al. designed a β-cell-mimetic closed-loop system by linking glucose-mediated depolarization by ectopic voltage-gated calcium channels to the expression of insulin to treat type-1 diabetes^31^.

Regulation of synthetic systems expressing channels for hormonal control to rectify type-1 diabetes has been achieved in various ways: by direct electrical stimulation^32^, opto-electronically (by light)^33^, or by temperature change^34^. In addition, *OxyR*, a positive regulator of hydrogen peroxide-inducible genes, was recently applied for bioelectronic signal transduction in *E. coli*^35^. Thus, efficient engineering of signaling cascades can provide tight control of cellular behavior in response to a specific inducer at a physiological concentration. Many of these genetic switches are triggered by small molecules such as antibiotics^36^, vitamins^37^, and food additives^38^. However, the possibility of developing a generalized platform using engineered cells for label-free detection of small molecules, such as hormones, has not been explored.

Here, we aimed to construct such a platform. Specifically, we designed a versatile bioelectronic interface (VIBE) that employs stable monoclonal cell lines with engineered signaling cascades to communicate with an electronic interface. The VIBE platform employs a three-electrode system in which the working electrode is modified with single-walled carbon nanotubes (SWCNTs) and seeded with stable engineered cells overexpressing a receptor of the target analyte. Stable ectopic expression of a specific target receptor in the native molecular context of human cells improves receptor–ligand interactions and results in a protein shielding effect that can be quantified via changes in the electrical output signal. A key advantage of our system is that the target ligand can easily be changed simply by replacing the cellular component with another appropriately engineered cell clone that overexpresses the relevant receptor. As proof-of-concept, we show that the VIBE platform can directly differentiate insulin or GLP-1 levels in blood samples from wild-type and diabetic model mice, and can distinguish fasting and postprandial states in humans.

## Results

### Electrode design and characterization

**Fig. 1:**
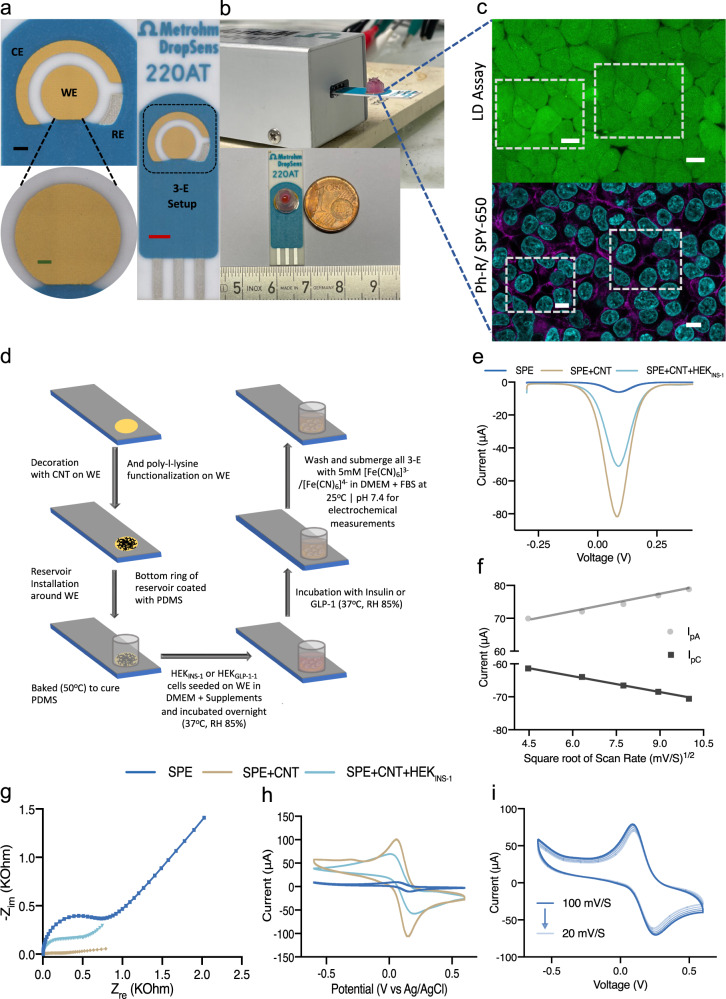
Schematic illustration, photographs, and electrode characterization of the VIBE platform. **a** Electrode dimensions, design (with top and side views), assembly, and electrochemical setup (Scale bars: red 2.5 mm, black 1 mm, green 500 µm) (CE counter electrode, WE working electrode, RE reference electrode, 3-E three electrodes). **b** A mesofluidic reservoir is used to segregate the WE from the Au-plated counter electrode and Ag/AgCl reference electrode. As shown, the chip is connected to the electrochemical workstation using a SPE connector. The reaction center or WE is smaller than a Euro cent. **c** Live-dead (LD) assay demonstrated cell viability (Green represents live cells) while performing electrochemical measurements (scale bar: 10 µm). Further, adhesion and morphology of the engineered cells on the surface of the working electrode were assessed by phalloidin-rhodamine (Ph-R)/ SPY-650 staining of the actin-myosin filaments (Magenta) and nucleus (Cyan), respectively (scale bar: 10 $\mu {{{{{\boldsymbol{m}}}}}}$). The square in the micrograph without a scale bar shows the enlarged area and the one with a scale bar shows the zoomed-in micrograph. Each of the experiments were repeated over three biologically independent samples and similar results were obtained from which a representative micrograph is portrayed in the figure. **d** Schematic illustration of electrode preparation, cell seeding, induction, and preparation procedures for electrochemical measurements. **e**, **g**, **h** Differential pulse voltammogram, impedance, and cyclic voltammetry-based characterization of the VIBE platform confirm that SWCNT modification increased the conductivity. After seeding the engineered cells, the peak current of the working electrode remained eight times higher than that of the bare SPE. **f** Electrical conductivity of the VIBE platform increases with the increasing square root of scan rate in cyclic voltametric characterization and satisfied the Randles–Sevcik equation for diffusion-controlled reactions in an electrochemical setup. **i** Increase in oxidation or reduction current proportionate with scan rate demonstrates the diffusion-based nature of the electrochemical reaction and confirms shifts of the anodic current towards higher potential while cathodic current to lower potential. Source data are provided as a Source Data file.

The VIBE system consists of a central gold (Au)-plated circular working electrode (WE) surrounded by a counter electrode (CE) and a reference silver electrode (RE) (Fig. 1a). The electrode was connected to the electrochemical workstation using an SPE connector (Fig. 1b). The surface of the chip was decorated with SWCNTs to enhance the conductivity (Fig. 1d, e). A schematic of the entire electrode preparation procedure is provided in Fig. 1d. Transmission electron microscopy (TEM) of SWCNTs revealed that the diameter of the nanotubes ranged from 0.8 to 1.2 nm (Supplementary Fig. 1a–c). PDMS, an FDA-approved gluing resin, was used to attach the mesofluidic reservoir to the periphery of the working electrode to isolate it from the counter and reference electrodes, and to provide a chamber for seeding engineered cells with their culture medium (Fig. 1d). The set-up of the VIBE platform and sensing unit is shown in Fig. 1b. Engineered human embryonic kidney cells (HEK_INS-1_) seeded on the sensing platform showed good compatibility with the SWCNT-modified electrode surface (Fig. 1c and Supplementary Fig. 2a), and their metabolic activity was similar to that of cells cultured in plates under standard conditions (Supplementary Fig. 3a, b). HEK_INS-1_ cells grown on the electrode surface retained regular morphology with an intact nucleus and actin-myosin filaments, as revealed by rhodamine and SPY-650 staining (Fig. 1c). Calcein AM- or ethidium homodimer (ETD-1)-based assays (ref. ^39^) confirmed good viability of the cultivated engineered HEK_INS-1_ cells during the electrochemical measurements (Fig. 1c).

The sensing principle of the VIBE platform is based on signal suppression of the system due to increased resistance upon the interaction of the agonist with its receptor. SWCNT decoration of the WE led to a significant increase in the final output current of SPE + SWCNT + HEK_INS_ as compared to the bare SPE even after cell seeding, thereby confirming that the SWCNTs were retained on the surface of the working electrode (Fig. 1e, h). Further, micrographs from field-effect scanning electron microscopy (FESEM) also confirmed CNT retention on the working electrode (Supplementary Fig. 4a–f). All the electrochemical measurements were performed in the presence of 5 mM [Fe(CN)_6_]^3-^/[Fe(CN)_6_]^4-^ redox couple, which acts as an electron transfer mediator enabling detection and characterization of the output current. Impedance analysis confirmed an increase in resistivity of the system (Fig. 1g). A linear increase in oxidation (I_pA_) or reduction (I_pC_) current with the increasing square root of scan rate satisfied the Randles–Sevcik equation for diffusion-controlled reactions in an electrochemical setup (Fig. 1f)^40^. An analysis of the electrical conductivity of the VIBE platform with increasing scan rate in cyclic voltametric characterization of the bioelectronic interface confirmed a shift of anodic current towards higher potential and of cathodic current towards lower potential due to a decrease in the probability of occurrence of the redox reactions at higher scan rate (Fig. 1i).

### Receptor density on the VIBE platform

The vectors carrying the genomic sequence encoding for human insulin receptor *(hIR)* (ITR-P_hCMV_-hIR-R-pA:P_hCMV_-ZeoR-P2A-mRuby-pA-ITR) in HEK_INS-1_ or *GLP-1R* (ITR-P_hCMV_-GLP-1R-pA:P_hCMV_-BlastR-P2A-iRFP-pA-ITR) in HEK_GLP-1-1_ co-express fluorescent labels, mRuby and iRFP, respectively (Supplementary Table 1). Fluorescence-activated cell sorting (FACS) demonstrated that 98.9% of HEK_INS-1_ were positive for mRuby and 98.3% of HEK_GLP-1-1_ cells were positive for iRFP, confirming efficient expression and distribution of hIR and GLP-1 receptors on the cells (Supplementary Fig. 5a, c). The cells containing these receptors mediate the on-chip detection of the hormones. We also performed a fluorescence microscopic analysis of the density of cell-surface receptors on cells seeded on top of the electrodes. The micrographs in Supplementary Fig. 7a, b show high fluorescence intensity of the fluorescent tags of the cell-surface receptors on the electrode surface, confirming superior on-chip distribution as compared with native HEK-293 cells (without plasmids) (Supplementary Fig. 7c) (negative control). Image analysis of the acquired fluorescence micrographs (Supplementary Fig. 7d) revealed significant orders of magnitude increase (117x and 50x for insulin and GLP-1 receptors, respectively) of the mean receptor density on the electrode surface compared with native HEK-293 cells.

### Insulin sensing by the VIBE platform (VIBE_INS_)

**Fig. 2:**
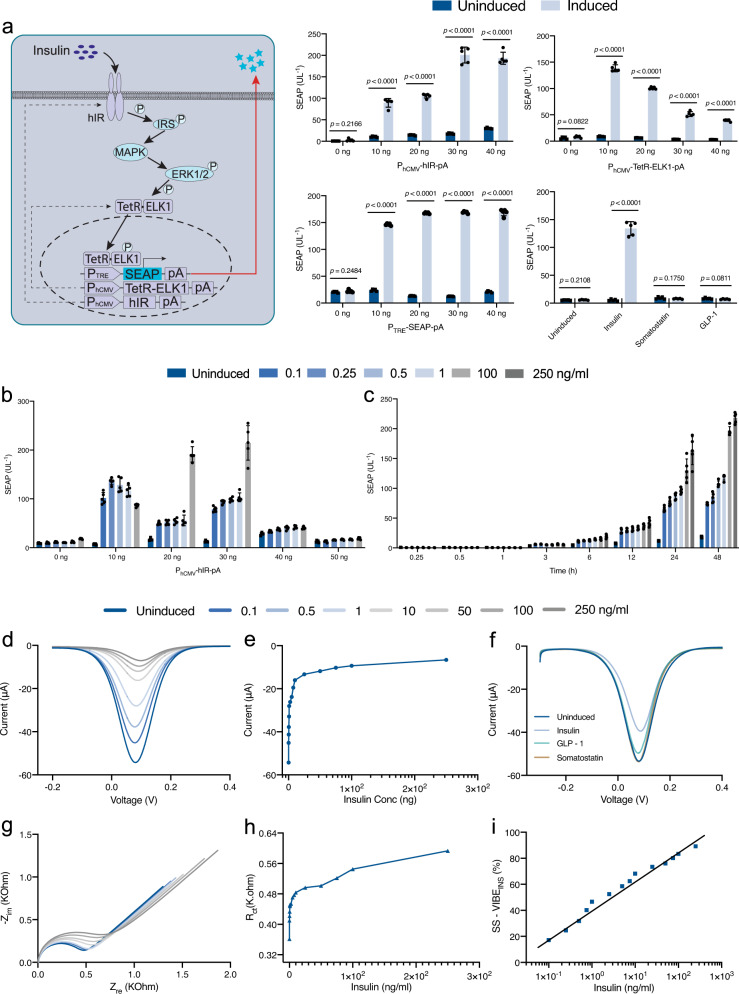
Engineering VIBE_INS_ and electrochemical detection of insulin with the VIBE_INS_ platform. The VIBE_INS_ platform utilizes HEK_INS-1_ cells. **a** A synthetic insulin-sensing cascade was built by overexpressing human insulin receptors (hIR) in the plasma domain. Activation of IR triggers autophosphorylation of multiple tyrosine residues, resulting in the activation of intracellular proteins such as insulin receptor substrate (IRS) proteins and MAPK. Phosphorylated MAPK phosphorylates TetR-ELK1, which then migrates into the nucleus and interacts with the tetO operator of a tetracycline-responsive promoter (P_TRE_), triggering the production and secretion of SEAP. Optimization of the ratio of plasmids phIR (P_hCMV_-IR-pA), MKp37 (P_hCMV_-TetR-ELK1-pA), and pMF111 (P_TRE_-SEAP-pA). The molar ratio of 3:1:3 was optimum for detecting 1 ng/ml of insulin. Non-specific interaction with competing hormones like somatostatin (0.1 ng/ml) and GLP-1 (0.1 ng/ml) was also tested. Data were presented as mean ± SD of *n* = 5, biologically independent samples. *p* value was calculated using a two-tailed, unpaired Student’s *t*-test. **b** Effect of plasmid transfection amount. 30 ng/well of P_hCMV_-IR-pA was optimum for receptor–ligand interaction. Data were presented as mean ± SD of *n* = 5, biologically independent samples. **c** Kinetic study of transiently transfected HEK_INS_ over 48 h. Data were presented as mean ± SD of *n* = 5, biologically independent samples. **d**, **e** Differential pulse voltammetry (DPV) analysis and calibration of VIBE_INS_ show a decrease in output current with increasing concentration of insulin throughout the screening range (0.1–250 ng/ml). **f** DPV study of specificity shows negligible signal suppression by non-specific analytes, GLP-1 and somatostatin. **g**, **h** Frequency resonance analysis (FRA) of the interaction between IR and insulin is represented by a Nyquist plot. The derived R_ct_ values indicate an increase in resistance of the VIBE_INS_ platform with increasing concentration of insulin, in accordance with the DPV analysis. **i** A log-plot of signal suppression (SS-VIBE_INS_(%)) with increasing concentration of insulin shows the VIBE_INS_ platform can achieve up to 78% suppression of the output signal. Source data are provided as a Source Data file. Color bars and lines represent the concentration of insulin (ng/ml) used for induction. (ng/ml nanogram/milliliter).

Human embryonic kidney cells (HEK-293) were engineered for ectopic expression of the human insulin receptor (IR, phIR, P_hCMV_-hIR-pA), which was rewired to the downstream native signaling cascade, the mitogen-activated protein kinase (MAPK) pathway, via insulin receptor substrate (IRS) (Ye et al., 2016). MAPK was rerouted through a synthetic transcription factor containing the tetracycline-dependent repressor (TetR) with its sequence-specific DNA binding domain fused to the mammalian *ETS transcription factor Elk1 (Elk-1)*, which was constitutively driven by human cytomegalovirus promoter (MKp37, P_hCMV_-TetR-Elk1-pA). To validate this construct, HEK-293 cells were co-transfected with phIR (P_hCMV_-hIR-pA), MKp37 (P_hCMV_-TetR-Elk1-pA), and pMF111 (P_TRE_-SEAP-pA), a reporter plasmid encoding human placental secreted alkaline phosphatase (SEAP) under the TetR-ELK1-specific tetracycline-responsive promoter (P_TRE_) (Fig. 2a). HEK-293 cells co-expressing all transgenic vectors for rewiring signal transduction from transmembrane insulin receptors were termed HEK_INS_.

SEAP assay confirmed that HEK_INS_ is highly specific for insulin (1 ng/ml), showing minimal response to normal culture medium or medium containing physiological concentrations of unrelated peptide hormones such as glucagon (0.1 ng/ml) or somatostatin (0.1 ng/ml). These results demonstrate negligible leakiness and high specificity of the synthetically engineered pathway (Fig. 2a). Resazurin assay confirmed high cell viability in all cases (Supplementary Fig. 3a). In this work, it was of paramount importance to optimize the ratios of the expression vectors to attain maximal performance of the HEK_INS_ cells in the presence of the receptor agonist insulin, and the molar ratio of 3:1:3 of the expression vectors phIR (P_hCMV_-IR-pA), MKp37 (P_hCMV_-TetR-ELK1-pA) and pMF111 (P_TRE_-SEAP-pA) provided best-in-class downstream signaling cascade-guided activation of the human insulin receptor system (Fig. 2a). HEK_INS_ cells transfected with 30 ng/well of P_hCMV_-IR-pA was optimum for receptor–ligand interaction in a 96-well plate format (Fig. 2b), and this clinically relevant concentration was used for further studies. The transcription-based expression of SEAP was detectable at 6 h after activation of the insulin receptor and increased time-dependently (Fig. 2c). A dose-dependence study confirmed that SEAP was significantly and concentration-dependently increased by insulin at 0.1 ng/ml and higher concentrations (Supplementary Fig. 8a).

We used the best-in-class combination of expression vectors identified above to generate monoclonal HEK_INS-1_ cell lines stably expressing the synthetically engineered cascade (Supplementary Fig. 9a, b). Quantitative PCR (qPCR) analysis showed that the ratio of expression levels among hIR, TetR-ELK1, and P_TRE_-SEAP-pA transgenes in the HEK_INS-1_ with best performance is around 1:6.5:1.2 (Supplementary Fig. 9c). HEK_INS-1_ cells were seeded onto the bioelectronic device, hereafter termed as VIBE_INS_, for on-chip ultrasensitive real-time detection of insulin. VIBE_INS_ was then exposed to insulin and differential pulse voltametric analysis of the output current was performed to examine the change in electronic behavior of the chip modulated by the receptor–agonist interaction. A gradual decrease in the output current was observed with an increase in receptor–ligand interaction (Fig. 2d and Supplementary Fig. 8b), suggesting a masking effect on the transfer of electrons for the redox couple [Fe(CN)_6_]^3−^/[Fe(CN)_6_]^4^, as expected. At a physiological concentration of insulin (0.5–1.0 ng/ml), the output current measured by differential pulse voltammetry was decreased significantly from the original value, suggesting excellent sensitivity of VIBE_INS_ (Fig. 2d, e). Glucagon and somatostatin caused negligible changes in the voltammogram, confirming that the signal suppression is due to specific receptor–ligand interaction, in agreement with the results of SEAP analysis (Fig. 2a, f). No significant difference in the viability of HEK_INS_ cells was observed before and after the electrochemical measurements (Supplementary Fig. 2b). Further, in an electrochemical measurement, the VIBE_INS_ platform was tested blind with random concentrations of the target analyte to confirm that the output current was dependent upon the hormone concentration (Supplementary Fig. 10a). In a separate assay, SEAP produced from HEK_INS-1_ upon induction with various concentrations of insulin was electrochemically detected using PNPP. After overnight incubation of the VIBE_INS_ platform with various concentrations of insulin, PNPP was added as described in the analytical assay section and incubated for 5 min at 37 ^o^C. Cyclic voltammetry analysis revealed an increase of the output current with increasing concentration of the inducer owing to the hydrolysis of PNPP to 4-hydroxylaminophenol in the presence of SEAP (Supplementary Fig. 11a). This reverse analysis corroborates the insulin recognition ability of the VIBE_INS_ platform. Finally, when sensor readings from multiple electrodes were recorded, an 8–10% standard deviation in output current was observed in sensor recordings from three different electrodes at different stages of fabrication and sensing (Supplementary Fig. 12a–d). Further, no significant difference in %SS values were observed among electrodes compared at the same inducing concentration of insulin (Supplementary Fig. 12e). These results are consistent with the reported findings^41^. The above data are consistent with the idea that specific interaction between insulin receptors and insulin produces a protein shielding effect that inhibits electron transfer, thus increasing the resistance of the system. The sensor records resistance to electron transfer in the redox couple [Fe(CN)_6_]^3−/4−^ at the surface of the working electrode owing to protein shielding^42^. Aydin et al. similarly used the increase in charge transfer resistance due to hindered diffusion of [Fe(CN)_6_]^3−/4−^ as a parameter to detect increasing concentrations of IL-1$\beta$ on an ITO-based electrochemical sensor^43^. Similarly, small molecule recognition by a DNA scaffold-based electrochemical sensor caused a significant reduction in acquired current^41^. Frequency response analysis (FRA) of the VIBE_INS_ interface was performed to determine the change in relative charge transfer resistance (R_ct_) as a function of insulin concentration. Based on an equivalent circuit for the Nyquist plot (Supplementary Fig. 13), we determined the R_ct_ value of the VIBE_INS_ interface at different insulin concentrations (Fig. 2g, h). The percent signal suppression (%SS), increased concentration-dependently, reaching up to 78% (Fig. 2i). A time-lapse video of HEK_INS-1_ cells during the measurement process was recorded, and live-dead staining confirmed the stability and viability of the HEK_INS-1_ cells (Supplementary Movie 1). A cell count analysis indicated no significant difference in cell number before and after the electrochemical measurements (Supplementary Fig. 2c).

### GLP-1 Sensing by the VIBE platform (VIBE_GLP-1_)

**Fig. 3:**
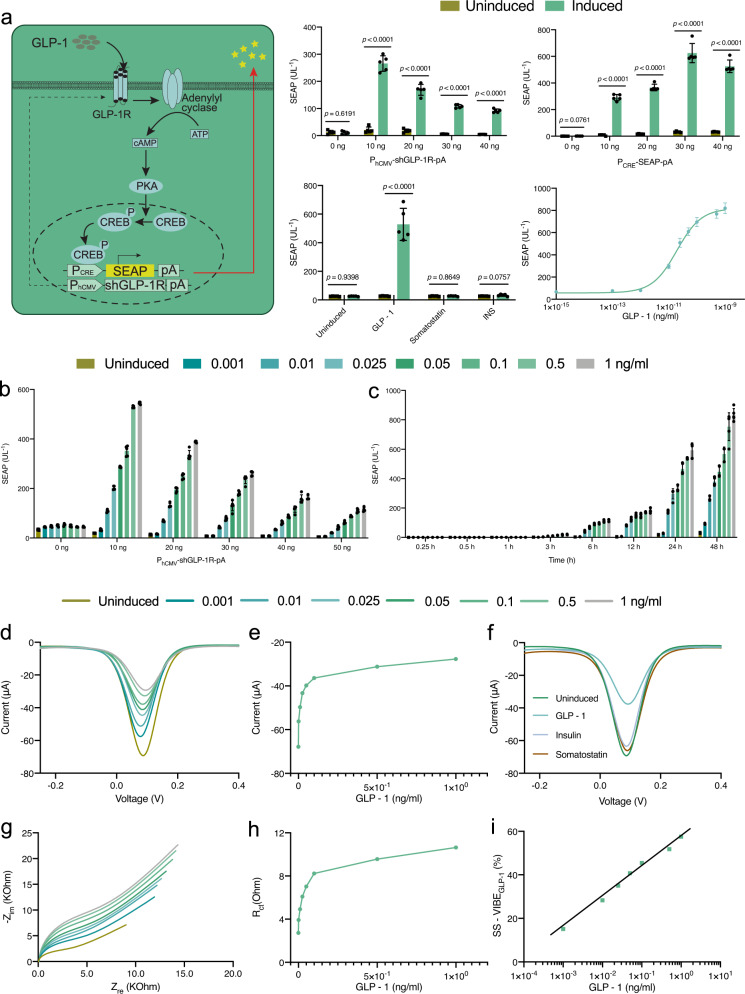
Engineering VIBE_GLP-1_ and electrochemical detection of GLP-1 with the VIBE_GLP-1_ platform. The VIBE_GLP-1_ platform employs HEK_GLP-1-1_ cells. **a** The synthetic GLP-1-sensing cascade was built by overexpressing GLP-1 receptors (pGLP-1R, P_hCMV_-shGLP-1R-pA) at the transmembrane domain and engineering a synthetic promoter (P_CRE_) coupled to a human placental secreted alkaline phosphatase (SEAP) reporter construct pCK53 (P_CRE_-SEAP-pA). The ratio of plasmids, P_hCMV_-shGLP-1R-pA and P_CRE_-SEAP-pA in 1:3 was optimized for transient transfection of HEK-293 cells in detecting 0.1 ng/ml of GLP-1. Non-specific interaction with competing hormones like somatostatin (0.1 ng/ml) and insulin (INS) (1 ng/ml) was also tested. Dose-response curve for activation of the genetic cascade by GLP-1R interaction with GLP-1. Activation occurs at 0.01 ng/ml and higher concentrations. Data were presented as mean ± SD of *n* = 5, biologically independent samples. *p* value was calculated using a two-tailed, unpaired Student’s *t*-test. **b** Optimization of receptor density on the transmembrane domain demonstrated 10 ng of P_hCMV_-shGLP-1R-pA /well in 96-well plate, gave the optimum receptor concentration. Data were presented as mean ± SD of *n* = 5, biologically independent samples. **c** Kinetic study of transiently transfected HEK_GLP-1_ showed a consistent increase in protein expression with increasing concentration and time over 48 h without saturation. Data were presented as mean ± SD of *n* = 5, biologically independent samples. **d**, **e** Differential pulse voltammetry (DPV) analysis and calibration of GLP-1 detection using VIBE_GLP-1_. The output current decreased with increasing concentration of GLP-1 throughout the physiological concentration range (0.01–0.1 ng/ml). **f** DPV shows negligible signal suppression by insulin or somatostatin, confirming high specificity for GLP-1. **g**, **h** Frequency resonance analysis (FRA) of the GLP-1–VIBE_GLP-1_ interaction is shown in a Nyquist plot and R_ct_ values show that the resistance of the VIBE_GLP-1_ platform increases with increasing concentration of GLP-1. **i** A log-plot of signal suppression (SS-VIBE_GLP-1_(%)) shows that suppression of the output signal with increasing concentration of GLP-1 reaches ~58%. Source data are provided as a Source Data file. Color bars and lines represent the concentration of GLP-1 (ng/ml) used for induction. (ng/ml nanogram/milliliter).

The developed VIBE interface was designed to be applicable for sensing a broad range of hormones simply by changing the engineered cells in the system. To test this idea, we set out to apply VIBE for sensing glucagon-like peptide-1 (GLP-1) by engineering human embryonic kidney cells (HEK-293) to ectopically express glucagon-like peptide-1 receptor (pGLP-1R, P_hCMV_-shGLP-1R-pA). GLP-1R was co-expressed with a reporter plasmid (pCK53, P_CRE_-SEAP-pA) containing a synthetic promoter (P_CRE_) to trigger expression of the SEAP transgene upon activation of the cAMP-CREB (cAMP, cyclic AMP; CREB, cAMP-responsive element-binding protein) signaling cascade (Fig. 3a), thus rewiring the complete signal transduction cascade upon activation of GLP-1R by GLP-1. GLP-1R is a G protein-coupled receptor (GPCR) and its activation by GLP-1 leads to the activation of membrane-bound adenylyl cyclase (AC), which converts ATP into cyclic AMP (cAMP). An increase in cytosolic cAMP activates protein kinase A, which translocates to the nucleus and phosphorylates cAMP-responsive element-binding protein 1 (CREB1). This binds to the synthetic promoter (P_CRE_), inducing P_CRE_-driven activation of the reporter construct (SEAP). Thus, the complete signal transduction cascade is rewired to respond to the activation of GLP-1R by GLP-1.

SEAP quantification demonstrated excellent specificity and successful activation of the engineered signaling cascade by GLP-1 with minimal background (Fig. 3a). Insulin and somatostatin had negligible activity (Fig. 3a). Resazurin assay confirmed similar viability of the cells in all experimental groups (Supplementary Fig. 3b). The optimum molar ratio of the expression vectors pGLP-1R (P_hCMV_-shGLP-1R-pA) and pCK53 (P_CRE_-SEAP-pA) was 1:3, with minimal background (Fig. 3a). A dose-dependence analysis confirmed activation of the receptor system at 0.01 ng/ml GLP-1, and the SEAP expression increased concentration-dependently (Fig. 3a). We also examined the effect of the level of receptor expression by varying the amount of plasmid used for transfection, and found that the receptor–ligand interaction was optimum at 10 ng of pGLP-1R (P_hCMV_-shGLP-1R-pA) (per well of a 96-well plate containing 1.2 × 10^5^ HEK-293 cells/ml) (Fig. 3b). A kinetic study showed that activation of the recognition system was significant and strong activation was seen from 6 h (Fig. 3c). SEAP expression levels continued to increase with time and ligand-concentration-dependently.

As noted above, a ratio of pGLP-1R (P_hCMV_-shGLP-1R-pA) and pCK53 (P_CRE_-SEAP-pA) of 1:3 was optimum, and was used to generate stable monoclonal cell lines (HEK_GLP1-1_ cells) for seeding onto our electronic interface (Supplementary Fig. 14a, b). qPCR analysis of the stable monoclonal lines revealed that the transgenes for GLP-1R and P_CRE_-SEAP-pA in HEK_GLP-1-1_, were expressed in the ratio of 1:0.8 (Supplementary Fig. 14c). HEK-293 cells transfected with the complete synthetic circuit, hereafter termed HEK_GLP-1-1_, were further incubated with GLP-1 alone or with insulin (1 ng/ml) or somatostatin (0.1 ng/ml) to confirm successful integration and specificity of the synthetic circuit (Supplementary Fig. 14b). The resulting bioelectronic device was termed VIBE_GLP-1_. Differential pulse voltammetry (DPV) was conducted to examine the dose-dependence of GLP-1 interaction with VIBE_GLP-1_, and a significant change in output current was observed from 0.001 ng/ml GLP-1; the output current decreased further in proportion to the increase of hormone concentration (Fig. 3d). The normal physiological concentration of GLP-1 in human blood ranges from 0.01 to 0.05 ng/ml in the fasting and postprandial states, respectively. A representative calibration plot for DPV is shown in Fig. 3e. Specificity analysis confirmed that VIBE_GLP-1_ showed negligible interaction with unspecific hormones (Fig. 3f). Blinded electrochemical measurement with the VIBE_GLP-1_ platform was also performed to confirm that the sensor’s output current was dependent on the GLP-1 concentration (Supplementary Fig. 10b). Electrochemical SEAP analysis of the VIBE_GLP-1_ platform confirmed that after overnight incubation with various concentrations of GLP-1, increasing levels of SEAP hydrolyzed PNPP to generate higher current with increasing concentration of the inducer, thus further reaffirming the recognition efficacy of the VIBE_GLP-1_ platform (Supplementary Fig. 11b). Protein shielding on VIBE_GLP-1_ was also analyzed using frequency response analysis (FRA) and R_ct_ values were obtained using an equivalent circuit (Supplementary Fig. 13). The R_ct_ values increased proportionally to GLP-1 concentration, confirming that the protein shielding effect on VIBE_GLP-1_ impairs transfer of electrons for the redox couple [Fe(CN)_6_]^3−^/[Fe(CN)_6_]^4−^ (Fig. 3g, h). A log-plot of signal suppression (%) indicated that suppression of the output signal reached around 57% with increasing concentration of GLP-1 (Fig. 3i). All these results suggest that the VIBE interface is versatile and can be easily modified to target a range of analytes based on specific receptor–ligand interaction.

### Storage of the sensors

Treating HEK_INS-1_ and HEK_GLP-1-1_ cells with a low concentration of mitomycin C (MMC) (1 µg/ml or lower) inhibited cell division while keeping the cells metabolically active for 10 days (Supplementary Fig. 15a, b)^44^. VIBE_INS_ or VIBE_GLP-1_ platforms deployed with MMC-treated cells were able to detect levels of insulin and GLP-1 at various time points up to 10 days (Supplementary Fig. 15c, d). The MMC treatment blocks over-confluency, enabling the sensing platforms to be stored at 37 ^o^C and RH 85% in supplementing medium for 10 days. Alternatively, since this is a segregated system, the two components can be stored separately, i.e., the engineered cells can be kept in liquid nitrogen and the electrode system can be kept in a vacuum-sealed or nitrogen-flushed environment for a long period. The cells can be revived when required and added to the pre-fabricated sensing platform for regular sensing.

### Validation of VIBEs for in vivo samples

**Fig. 4:**
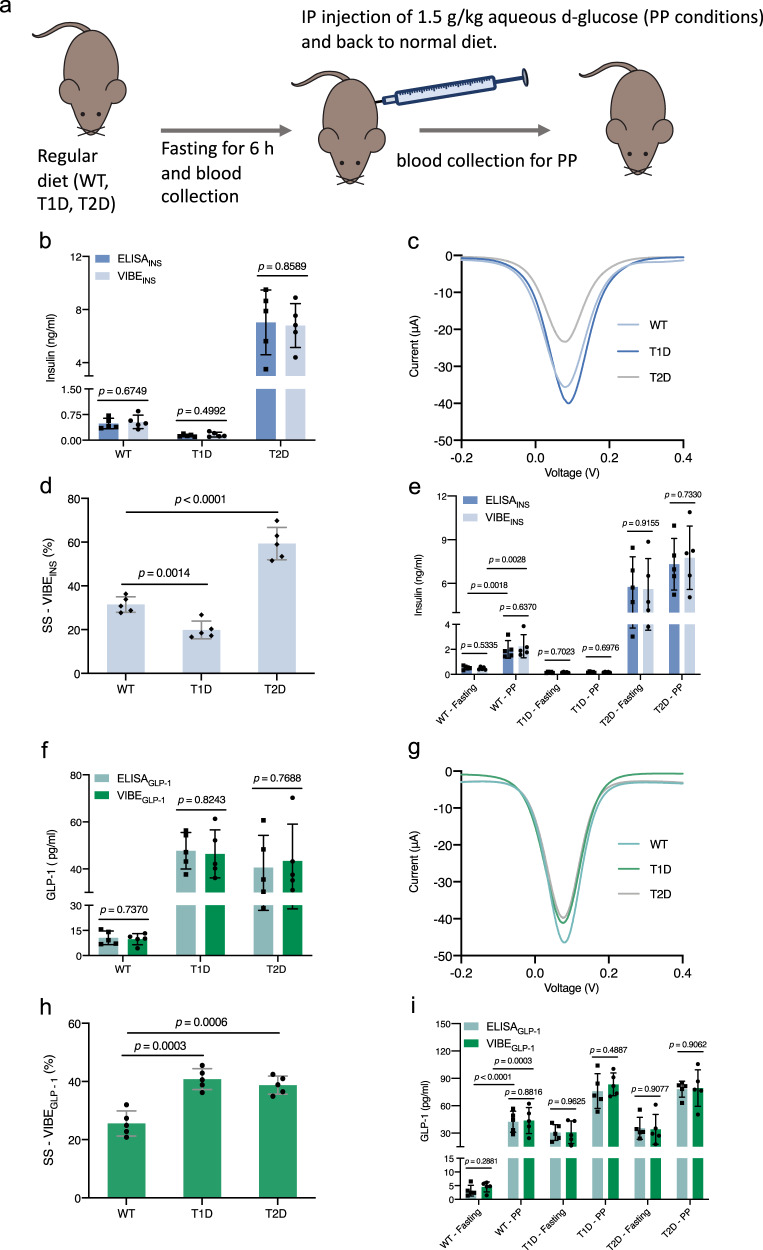
Analysis of mouse blood samples using the VIBE_INS_ and VIBE_GLP-1_ platforms. **a** A schematic of the experimental design. Blood samples from wild-type mice, experimental type-1 diabetic mice and type-2 (db/db) diabetic mice were collected under normal feeding conditions to test the response of the engineered platforms. Further, these mice were fasted for 6 h, followed by injection with 1.5 g/kg aqueous d-glucose to induce a postprandial-mimicking condition, and blood samples were collected. **b**–**d**, **f**–**h** The VIBE_INS_ and VIBE_GLP-1_ platforms can differentiate insulin and GLP-1 concentrations, respectively, in the three types of mice. This is also evident from the DPV plots. The results are consistent with those obtained by ELISA. The values for WT mice were significantly different from those of type-1 or type-2 diabetic mice. For **b** and **f**, data were presented as mean ± SD of *n* = 5, biologically independent samples and *p* value was calculated using a two-tailed, unpaired Student’s *t*-test. For **d**, **h**, data were presented as mean ± SD of *n* = 5, biologically independent samples and *p* value was calculated using a two-tailed, unpaired Student’s *t*-test. **e**, **i** VIBE_INS_ and VIBE_GLP-1_ can differentiate insulin and GLP-1 concentrations, respectively, under fasting and postprandial conditions for wild-type mice and the values were in accordance with those of ELISA assays. For **e**, **i**, data were presented as mean ± SD of *n* = 5, biologically independent samples and *p* value was calculated using a two-tailed, unpaired Student’s *t*-test. Source data are provided as a Source Data file. ng/ml nanogram/milliliter, pg/ml picogram/milliliter. SS-VIBE_INS_(%) and SS-VIBE_GLP-1_(%) represent signal suppression (%) of VIBE_INS_ and VIBE_GLP-1_, respectively upon induction.

We next examined the potential utility of the VIBE platform as a point-of-care device by applying it to blood samples from wild-type mice, type-2 diabetic (db/db) mice, and experimental type-1 diabetic mice (Fig. 4a). VIBE_INS_ or VIBE_GLP-1_ could effectively differentiate these mice (Fig. 4b, f). Thus, signal suppression (SS %) and the DPV plots for VIBE_INS_ or VIBE_GLP-1_ can be utilized as a quantitative indicator of the level of insulin or GLP-1 in blood samples, and should be available as a parameter for future point-of-care devices (Fig. 4c, d, g, h). To further validate the device’s performance, a gold-standard enzyme-linked immunosorbent assay (ELISA) was performed in each case, and good agreement was observed between the hormone levels quantified by the two methods. This implies that naturally occurring serum proteins do not interfere with sensing and highlights the specificity of the system, in accordance with the in vitro evaluation. Encouraged by these results, we performed a classic metabolic test. Mice from the three groups were fasted for 6 h and then injected intraperitoneally with 1.5 g/kg aqueous d-glucose (IP) to mimic a postprandial state. VIBE_INS_ and VIBE_GLP-1_ could quantify insulin and GLP-1, respectively, in both fasting or postprandial states (Fig. 4e, i). The VIBE_INS_ platform could efficiently detect the postprandial surge in insulin concentration in wild-type mice, and clearly differentiated the non-significant insulin surge in experimental type-1 or type-2 diabetic mice (Fig. 4e). As regards GLP-1, a significant postprandial excursion of GLP-1 levels was detected in wild-type mice, as expected^1^ (Fig. 4i). Higher basal GLP-1 expression was observed in experimental type-1 diabetic and type-2 diabetic (db/db) mice, but a postprandial change of GLP-1 levels was still detectable by the VIBE_GLP-1_ platform (Fig. 4i). The calibration curve plots used for determining concentrations of insulin and GLP-1 from DPV plots are shown in Supplementary Fig. 16a, b.

### Analysis of human serum samples

**Fig. 5:**
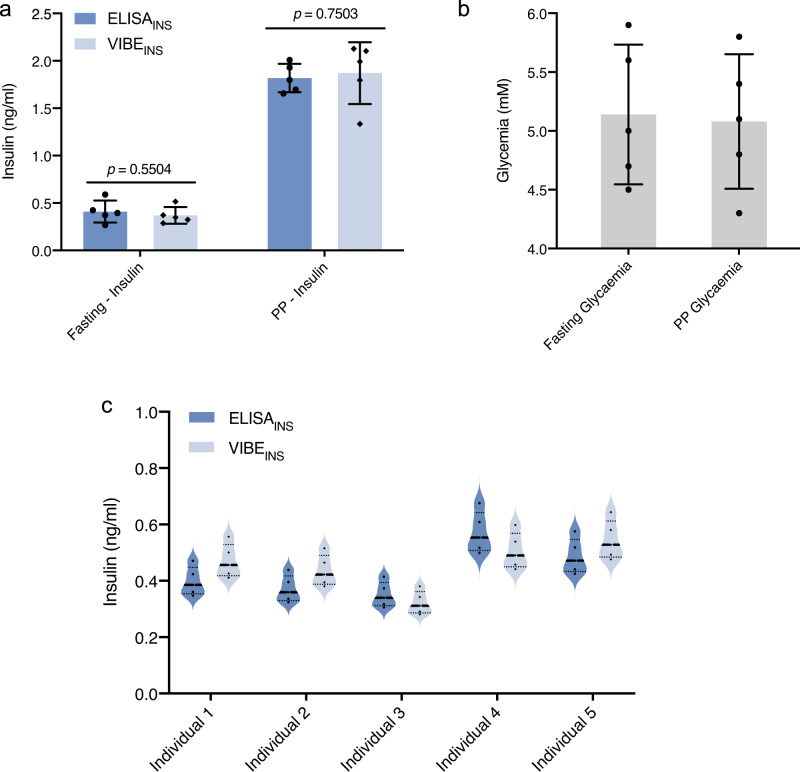
Evaluation of human blood samples using the VIBE_INS_ platform. Blood from healthy individuals were collected and insulin was measured using the VIBE_INS_ platform. **a**, **b** Serum insulin in the postprandial (PP) state is significantly higher than in the fasting condition, suggesting effective islet function, thus leading to normoglycemic condition at PP. For **a**, **b**, data were presented as mean ± SD of *n* = 5, biologically independent samples. For a, *p* value was calculated using a two-tailed, unpaired Student’s *t*-test. **c** Insulin levels in the serum of different individuals (*n* = 5) in a normal metabolic condition were within the expected range. Insulin levels in all the samples were also measured by ELISA and there was no significant difference between the ELISA and VIBE_INS_ results. Source data are provided as a Source Data file. ng/ml nanogram/milliliter, mM millimolar.

To investigate potential clinical applicability, blood samples were collected from healthy individuals of various origins, ages, and dietary habits under fasting or postprandial conditions. We focused on insulin owing to its direct relationship with diabetes. The VIBE_INS_ platform detected a significant postprandial elevation of insulin levels (Fig. 5a), but the glycaemic levels did not show large excursions, consistent with normal metabolism (Fig. 5b). Repeated analyses using VIBE_INS_ showed no significant difference from values obtained with clinically approved ELISA assays, confirming the accuracy and reproducibility of the developed platform (Fig. 5c).

## Discussion

Previous attempts to develop an interface between electronic and genetic circuits have not yet met the need for a versatile and robust platform sensitive to changing concentrations of small molecules such as hormones. Here, we designed, constructed, and validated the VIBE platform, which employs monoclonal engineered cells stably expressing receptors connected to synthetic signaling pathways. The engineered cells not only provide a high density of receptors for the capture of the target ligand, but also can be easily immobilized on the electrode surface while maintaining a stable receptor conformation (Supplementary Fig. 5). Importantly, the analytical target can be changed as required simply by replacing the engineered cells seeded on the working electrode of the VIBE system with other cells expressing the appropriate receptor. The SWCNTs decorating the working electrode facilitate the ballistic transport of electrons through the backbone, increasing the conductivity of the sensing platform (ref. ^45^). Further, the downstream signaling cascade increases the efficiency of the receptor–ligand interaction, because cells expressing the entire system showed higher signal suppression than cells expressing only the receptors (Supplementary Fig. 17). The human insulin receptor (hIR) possesses a tetrameric structure consisting of two extracellular α subunits and two transmembrane β subunits. When insulin (the ligand) interacts with the α subunits, hIR undergoes a conformational change to activate the kinase activity of the β subunits. Transphosphorylation between the β subunits leads to the recruitment of intracellular insulin receptor substrate (IRS) and the IRS acts as a scaffold for downstream signaling complexes^46,47^. The interaction of IRS protein activates the receptor through pleckstrin homology (PH) and phosphotyrosine binding (PTB) domains^48^. In a study by Araki et al., IRS-1 knockout (KO) mice demonstrated impaired insulin response^49^. Further, Withers et al. showed that IRS-2 KO mice exhibit defective insulin signaling along with loss of β cells, leading to diabetes^50^. Close to 78 and 57% suppression was observed in the presence of insulin or GLP-1, respectively, and the limit of detection was in the sub-picomolar range, which is one order of magnitude lower than those of reported systems (10–20 nM)^20,21^. The VIBE_INS_ and VIBE_GLP-1_ platforms both started to respond within the first 0.5 to 1 min, and significant increases in %SS values were obtained within 10 and 5 min, respectively (Supplementary Fig. 18a, b). The response times of the sensors are in line with those of existing insulin sensors^20,51^. The engineered cells express new receptors and transcription factors continuously. Indeed, when the sensors were used repeatedly at intervals of 1 h, they could be reused multiple times (Supplementary Fig. 18c, d) without any treatment with chemicals or external agents. Engineered cells ectopically expressing a high density of a desired receptor are also favorable for achieving appropriate receptor orientation and reproducible functionalization of the sensing platform, in contrast to antibody-functionalized platforms^24,26^. This signaling cascade-based detection with an electronic interface offers very high specificity and ultrasensitive detection of receptor–ligand interaction over a wide concentration range, which would be appropriate for practical and clinical applications (Figs. 2d, i, 3d, i). Indeed, the VIBE system could differentiate wild-type, experimental type-1 diabetic and type-2 diabetic (db/db) mice, and could also distinguish between fasting and postprandial conditions. In human samples, the interface could reliably detect a range of insulin concentrations corresponding to individuals’ insulin resistance and timing of food intake. Its high sensitivity also enabled the VIBE_INS_ system to detect even the low insulin levels of type-1 diabetic patients with residual insulin secretion. Furthermore, the small size of VIBE devices would be favorable for their deployment in implantable devices to sense and control physiological states in cell-based therapies via the internet of things. Multiplexing of VIBE_INS_ and VIBE_GLP-1_ could be an effective alternative to existing glucometers for analyzing a patient’s health and programming personalized medical interventions.

## Methods

### Ethics overview

In animal experiments to keep standard metabolic activity, and avoid the effect of heredity, breed, age, sex or weight, and following experimental conditions of previous reported literatures^32,52^, we selected 6- to 7-week-old male wild-type C57BL/6JRJ mice (average weight ~19–21 g, purchased from Janvier Labs) and 6- to 7-week-old male type-2 diabetic, BKS-Leprdb/db/JOrlRj mice (average weight ~ 30–35 g, purchased from Janvier Labs). Mice were maintained in groups and housed in a temperature (21 ± 2 °C) and humidity (55 ± 10%) controlled room with a 12 h inverse daylight cycle with free access to standard diet and drinking water. Animals of each type were divided randomly into different experimental groups. All animal experiments were performed in accordance with the Swiss animal welfare legislation and approved by the Veterinary Office of the Canton Basel-Stadt, Switzerland (license number: 2996_34477) and conducted by P.G.R (license number: LTK 5507) at the Department of Biosystems Science and Engineering (D-BSSE) of the ETH Zurich in Basel, Switzerland. For Human blood collection, all volunteers participating in this study were members of our research team and the collection procedure was performed by a medical practitioner, Dr. Henryk Zulewski (MD, doctor). The Ethics Committee of Northeastern and Central Switzerland (EKNZ) has classified the use of blood samples by healthy volunteers as a quality assurance project (09.079 message to the Federal law on research on human; https://www.fedlex.admin.ch/eli/fga/2009/1423/de) that is not within the scope of the Swiss Federal Act on research involving humans and does therefore not require formal approval by the ethics committee (project-ID: Req-2023-00581—evaluation of blood–insulin and glucose using a bioelectronic interface) based on the stipulations by the Swiss Federal Coordination Office for Human Research (https://www.kofam.ch/en/applications-and-procedure/projects-that-do-not-require-authorisation). Written consent was provided by all blood donors and none of them received any kind of compensation. The participants were from varying origins, ages, and dietary habits, and all gave prior written consent for volunteering in the blood collection procedure, and publishing any relevant data.

### Molecular cloning and DNA constructs

Details of the design and construction of the expression vectors are provided in Supplementary Table 1.

### Cell culture and transient transfection

#### Cell culture

Human embryonic kidney cells (HEK-293T, ATCC: CRL-3216) were cultured in Dulbecco’s modified Eagle’s medium (DMEM; Life Technologies, Carlsbad, CA, USA) supplemented with 10% (v/v) fetal bovine serum (FBS; Sigma-Aldrich, Munich, Germany) and 1% (v/v) penicillin/streptomycin solution (PS: Sigma-Aldrich, Munich, Germany) in a humidified atmosphere (relative humidity (RH) 85%) of 5% CO_2_ in air at 37 °C. Cell density were assessed using brightfield optics and a DirectPipette™-based device (CellDrop, Labgene Scientific SA, Châtel-Saint-Denis, Switzerland).

#### Transfection

Transfection of HEK-293 cells was performed according to a standard protocol in a 96-well plate format. A transfection mixture containing 200 ng of total plasmid DNA in 50 µl of reduced-serum Opti-MEM media (Gibco^TM^, USA), and 0.5 µl of polyethyleneimine (PEI; Polysciences Inc., Warrington, USA; 1 mg ml^−1^, DNA:PEI = 1:2.5) was prepared, vortexed, and incubated at 20 °C for 20 min before use. HEK-293 cells were seeded 15 h prior to transfection at a density of 1.2 × 10^5^ cells/ml. After the addition of 50 µl of transfection mixture, the plates were centrifuged at 200×*g* for 1 min and incubated under the above conditions for 12 h. Then, the supernatant from the transfected plates were replaced with fresh DMEM supplemented with FBS and the relevant inducer (insulin or GLP-1). After 24 h, the supernatant was collected and analysed for expression of SEAP.

### Stable cell line generation

HEK-293 cells were seeded at a density of 5 × 10^4^ cells/well in a 24-well plate. The cells were co-transfected with pJH2026 (ITR-P_hCMV_-hIR-pA:P_hCMV_-ZeoR-P2A-mRuby-pA-ITR), pJH2024 (ITR-P_hCMV_-TetR-ELK1-pA:P_RPBSA_-eCFP-P2A-PuroR-pA-ITR), pJH2025 (ITR-P_TRE_-SEAP-pA:P_hCMV_-BlastR-P2A-iRFP-pA-ITR) at the molar ratio of 3:1:3 for the insulin receptor system, while for GLP-1 receptor system, plasmids pJH2023 (ITR-P_hCMV_-GLP-1R-pA:P_hCMV_-BlastR-P2A-iRFP-pA-ITR), pJH2022 (ITR-P_CRE_-SEAP-pA:P_RPBSA_-eCFP-P2A-PuroR-pA-ITR) were co-transfected at the molar ratio of 1:3. Sleeping Beauty transposase expression vector pJH42 (P_hCMV_-SB100X-pA) (30 ng) was added to the transfection mixture in both cases. Cells transfected with the GLP-1 receptor system were cultivated in a medium containing 2 µg/ml puromycin and 10 µg/ml blasticidin, while for the insulin receptor system, 100 µg/ml of zeocin was also added along with the other two antibiotics. Both cell lines were cultivated for 2 weeks in a resistance medium, and stable clones of HEK_INS-1_ and HEK_GLP1-1_ were selected based on fluorescence intensity by means of fluorescence-activated cell sorting (FACS; FACSAria Fusion Cell Sorter, Becton Dickinson, New Jersey, USA). For FACS, a polyclonal population of engineered HEK-293T cells (1 × 10^6^ cells/ml) was suspended in FACS sorting buffer (PBS with 0.2% FBS) and filtered in a 5 ml polystyrene tube with a cell-strainer cap (35 µm, Falcon #352235) prior to sorting. Cells were kept on ice before the experiment. They were sorted into two different sub-populations positive for both mRuby and iRFP (HEK_INS-1_) and for both iRFP and eCFP (HEK_GLP-1_). The stable clones in Q2 of Supplementary Fig. 5b (positive for both mRuby and iRFP) for HEK_INS-1_ and Supplementary Fig. 5d (positive for both iRFP/eCFP) for HEK_GLP-1-1_ were selected and cultured in resistance medium to maintain their transgenicity. The gating strategies are shown in Supplementary Fig. 6a–h. The clones were periodically checked for contamination with mycoplasma or bacteria. FlowJo 10.5 software was used to analyse the FACS data.

### RNA sample extraction and quantitative PCR (qPCR) assay

The selected monoclonal cell lines were cultured overnight in 10-cm dishes. Then the cells were collected for total RNA extraction using a Quick-RNA Miniprep Kit (Zymo Research, cat. no. R1054), followed by quality and quantity control with a NanoDrop 2000 (Thermo Fisher). The cDNA library was prepared using a High-Capacity cDNA Reverse Transcription Kit (Applied Biosystems, cat. no. 4368814). The qPCR assay was analyzed by QuantStudio 3 (Thermo Fisher) using SsoAdvanced Universal SYBR Green Supermix (Bio-Rad, cat. no. 1725271). The primers used for GLP-1R, SEAP, hIR, and TetR-ELK1 and a house-keeping gene (glyceraldehyde 3-phosphate dehydrogenase, GAPDH) are listed in Supplementary Table 2 and were purchased from Sigma.

### Image analysis

Briefly, we segmented each image from each ND2 file into foreground fluorescence signal vs. background using the iso data algorithm implementation from scikit-image after a low-pass filtering step using a Gaussian kernel with sigma = 1.0 to suppress noise, and we then calculated and recorded the mean signal intensity of the foreground (the cells). Finally, for each group, we compared the mean intensities across ten images.

### Analytical assays

#### SEAP assay

Human placental secreted alkaline phosphatase (SEAP) levels in induced or uninduced cell cultured media were quantified by colorimetric assay. The cell culture supernatant was inactivated at 65 °C for 30 min. SEAP analysis mixture consisted of 80 µl of 2x SEAP assay buffer (20 mM homoarginine, 1 mM MgCl_2_, 21% diethanolamine, pH 9.8), and 20 µl of SEAP substrate solution (120 mM p-nitrophenyl phosphate, cat. no. AC128860100, Thermo Fisher Scientific, USA). The SEAP analysis mixture was added to the heat-inactivated cell culture supernatant in a ratio of 1:0.8 and the absorbance was recorded over 30 min at 405 nm and 37 °C using a plate reader (Tecan SPARK Reader; Tecan, Männedorf, Switzerland). The SEAP levels were analysed from a standard curve^53^.

#### Cell viability

To determine cell viability, the cells were incubated for 2 h in fresh DMEM supplemented with FBS and PS in the presence of 50 µg/ml resazurin (cat. no. R7017, Sigma-Aldrich, Saint Louis, MO, USA) under the standard conditions (85% RH, 5% CO_2_, 37 °C). Absorbance was measured at 540/590 nm using a plate reader (Tecan SPARK Reader, Männedorf, Switzerland).

#### Fluorescence microscopy

The cells were washed with 1x PBS three times and fixed using 4% paraformaldehyde solution in 1x PBS (pH 7.4) for 25 min at 37 °C^54^. After fixation, the cells were washed three times in 1x PBS and the cytoskeleton was stained using phalloidin-rhodamine (1x dilution, Abcam, Boston, USA, cat. no. ab235138, lot. no. GR3408497-2) for 25 min at 25 °C. The fixed cells were washed three times in 1x PBS and the nuclei were stained with SPY 650—DNA (1x dilution, Spirochrome, Thurgau, Switzerland, cat. no. SC501) for 45 min at 25 °C. Excess dye was removed by washing the cells twice with 1x PBS, and the cells were examined with a Zeiss LSM 980 fluorescence microscope (Carl-Zeiss, Oberkochen, Germany; Phalloidin-rhodamine excitation (Ex)/emission (Em) 546/575 nm, Spy 650—DNA Ex/Em, 652/674 nm). For live cell imaging (live-dead assay), the cells were washed with 1x PBS, one to three times and stained with a live-dead assay kit (4 mM Calcein AM, 2 mM EthD-1, Invitrogen, Thermo Fisher Scientific, Oregon, USA, cat. No. L3224, lot. no. 2261453) for 20 min under normal cell culture condition. After incubation, the cells were washed three times in 1x PBS and imaged at Ex/Em 450/490 nm (green, Calcein AM) and Ex/Em 510/560 nm (red, ETD-1) using a Zeiss LSM 980 fluorescence microscope. All micrographs were digitally processed using ZEN Blue software (Carl-Zeiss, Oberkochen, Germany; version. 3.5).

#### Electron microscopy

For TEM, the samples were coated on a Cu TEM grid and micrographs were acquired using STEM – JEOL JEM-F200 cFEG. For FESEM, samples were sputter coated with platinum (3–3.5 nm) using LEICA EM ACE 600 and microscopy was performed using HITACHI S-4800 scanning electron microscope.

### Chip design

We adopted a three-electrode screen-printed electrode design (SPE) (DRP-220AT screen-printed gold electrode, Metrohm, Suisse SA, Zofingen, Switzerland) consisting of gold-plated (Au) counter and working electrodes with a conventional silver electrode as the reference electrode. Prior to further modification, the SPEs were washed thoroughly by step-wise consecutive ultrasonication (Bioruptor, Diagenode; Seraing, Belgium) in acetone, ethanol, and double-distilled water (D_2_I) for 5 min each to eliminate any physically adsorbed impurities. They were immediately dried using nitrogen gas and stored in an air-tight vacuum desiccator prior to further modification. Purified single-walled carbon nanotubes (SWCNTs, Sigma, cat no. 773735, Saint Louis, MO, USA) (0.5 mg/ml) were dispersed in a cocktail of D_2_I containing 0.2 M *N*-(3-Dimethylaminopropyl)-*N*′-ethylcarbodiimide hydrochloride (EDC) (cat. no. E7750, Sigma-Aldrich) and 0.05 M *N*-hydroxysuccinimide (NHS) (cat. no. 130672, Sigma-Aldrich) and homogenized by ultrasonication for 10–15 min^55^. The cleaned SPEs were pre-heated at 80 °C followed by surface modification of the working electrode with 20 µl of SWCNT dispersion. The SWCNT-modified SPEs were dried at 60 °C for 1 h and washed three times with D_2_I to remove unbound SWCNTs. The working electrode was further modified with 20 µl of poly-l-lysine (cat. no. P4832, Sigma-Aldrich) and incubated for 1 h prior to washing the chips, three times with D_2_I. A mesofluidic reservoir (100 µl) was fitted over the circumference of the surface-modified working electrode (WE) using a clinically biocompatible FDA-approved resin, PDMS (SYLGARD® 184, Sigma-Aldrich, Cat. No. 761036), to prevent possible spillage of engineered cells, medium or biomolecules onto the counter and reference electrodes (Fig. 1b, d). PDMS and curing agent were mixed according to the manufacturer’s protocol and a thin coating was applied on the periphery of the bottom ring. The whole device was baked at 50^o^C to cure the PDMS and fix the reservoir on the circumference of the WE (Fig. 1d). Stably transgenic HEK_INS-1_ or HEK_GLP1-1_ cells were directly re-seeded onto the WE of the bioelectronic chip at a density of 4 × 10^5^ cells/ml in fresh DMEM containing FBS and antibiotics^32^. The cells were maintained in DMEM high-glucose medium (4.5 mg/dl glucose concentration) supplemented with antibiotics (blasticidin 10 µg/ml, puromycin 2 µg/ml, and zeocin 100 µg/ml) as required depending on the cell type, together with fetal bovine serum (FBS). The use of a resistance medium ensures maintained expression of the synthetic circuit in HEK_INS-1_ or HEK_GLP1-1_. After overnight incubation, the bioelectronic chips were used for the electrochemical detection of insulin and GLP-1. The viability, distribution of live/dead cells, and morphology analysis of the seeded cells on the chip surface before and after electrochemical measurements were studied using resazurin assay, live-dead assay, and phalloidin-rhodamine/SPY650 staining, respectively, as described above.

### Electrochemical experimentation

All electrochemical experiments were carried out using a multichannel CHI 760E electrochemical workstation (CH Instruments, Texas, USA). The modified electronic chips were connected to the CHI electrochemical workstation using a connector for screen-printed electrodes (DRP-DSC, Metrohm, Suisse SA). Before the experiment, the culture medium in the chips was replaced with high-glucose DMEM (supplemented with FBS) containing insulin (cat. no. I2643, lot. no. SLCG6865, Sigma-Aldrich) or GLP-1 (cat. no. G3265, lot. No. SLCF6873, Sigma-Aldrich) and the chips were incubated for various times at RH 85% and 5% CO_2_ to promote ligand-receptor interaction. After incubation, the chips were washed three times and medium in the chips was replaced, and all three electrodes of the sensing system were exposed to DMEM supplemented with FBS containing 5 mM redox couple potassium ferricyanide (cat. no. 702587, lot. no. MKCK2092, Sigma-Aldrich) [Fe(CN)_6_]^3−^/[Fe(CN)_6_]^4−^ to facilitate the electrochemical measurements. Electrochemical detection was carried out using differential pulse voltammetry (DPV) and frequency response analysis (FRA). Various concentrations of insulin and GLP-1 were tested to explore the performance of the device. DPV was conducted in the range of −0.2 to 0.4 V (Incr = 0.001 V, amplitude 0.05 V, pulse width 0.05 s, pulse period 0.5 s, sampling width 0.0167, sensitivity range 0.00001 (A/V)) while FRA analysis was done in the range between 0.1 to 1000000 Hz (Bias potential ~0.02 V and amplitude ~0.02 V). The percent signal suppression (%SS) values were derived from the original current values using the following Eq. (1):

1 $$\%{SS}=\frac{{Iu}-{Ii}}{{Iu}}x\,100$$

I_u_ is the initial current value in the uninduced condition and I_i_ is the output current in the induced condition.

### Mitomycin C (MMC) treatment

HEK_INS-1_ and HEK_GLP-1-1_ cells were seeded at a density of 1 × 10^5^ cells/well in a 12-well plate. After overnight incubation, the cell culture medium was replaced with fresh cell culture medium containing 1 µg/ml MMC (Sigma-Aldrich, cat. no. M5353) in DMEM supplemented with 10% FBS and 1% penicillin-streptomycin and incubated for 2 h. Then, the medium was replaced and the cells were washed in 1x PBS (3x) and resuspended in fresh cell culture medium (DMEM supplemented with 10% FBS and 1% penicillin-streptomycin). The cell viability test and electrochemical measurements were performed as described before.

### Animal experiments

#### Preparation of experimental mouse models

Wild-type (WT) mice, experimental type-1 diabetic (T1D) mice, and type-2 diabetic (T2D) (BKS-Leprdb/db/JOrlRj) mice were used. Experimental T1D mice were prepared by intraperitoneally (IP) injecting 8- to 9-week-old male wild-type C57BL/6JRJ mice (~20–25 g), consecutively for 4 days with streptozotocin (STZ; 50 mg/ kg, 0.2 M citrate buffer, pH 4.2; cat. no. S0130, Sigma-Aldrich) after fasting for 6 h (ref. ^56^). As control WT mice, C57BL/6JRJ mice (25 g, Janvier Labs) received similar injections without STZ under the same conditions. After 10 days, both groups were tested for persistent fasting hyperglycemia using clinically licensed Contour®Next test strips and the Contour®Next ONE reader (Ascensia Diabetes Care, Basel, Switzerland; cat. nos. 84191451 and 85659367, respectively) to confirm the T1D status of the STZ-treated group.

#### Blood collection

For initial analysis, blood was collected under normal physiological conditions from all groups. Further, mice from all groups were fasted for 6 h, followed by feeding and injection of 1.5 g/kg aqueous d-glucose (cat. no. G7021, lot. no. SLCK9341, Sigma-Aldrich) to mimic a postprandial (PP) condition. Blood was collected from the tail vein of fasting and PP mice in Microtainer® serum separator tubes. Serum was obtained by centrifugation (10 min, 6000×*g*, 4 °C; cat. no. 365967, Becton Dickinson, New Jersey, USA) and stored at −20 °C for further analysis. The electronic chip was incubated with 20 µl of serum for 15 min, then washed three times with 1x PBS. Electrochemical detection of insulin was performed using DPV in a medium containing 5 mM potassium ferricyanide solution. Insulin levels in serum were also quantified using an ultrasensitive ELISA assay (for insulin, cat. no. 10-1247-01, Mercordia, Uppsala, Sweden; for GLP-1, cat. no. 10-1278-01, Mercordia).

#### Blood collection from human subjects

The participants were divided into two groups. One group of five participants (four male, one female, aged between 28–35, mean age—29) took the metabolite challenge test in which blood was collected in the morning before and 1 h after food intake to observe insulin levels under fasting or PP conditions, respectively. Another group of five different individuals (five males, aged between 34–60, mean age—40.2) participated in the random blood–insulin level test. The lower mean age group took the metabolite test to stimulate an appropriate increase in blood–insulin levels, thus leading to blood glucose homeostasis at postprandial conditions.

The age group with higher mean age took the random blood–insulin level test. All blood samples were collected on the same day in heparin-coated tubes and centrifuged at 7500×*g* for 10 min to obtain serum. The VIBE_INS_ platform was incubated with the isolated serum, followed by thorough washing, and electrochemical measurements were performed as before. In the first set, individual fasting and PP samples were tested on separate VIBE_INS_ platforms, while for the second set each of the five separate samples was tested on five individual VIBE_INS_ platforms using the same vial of blood. The serum samples were also screened using a standard Human Insulin ELISA kit (cat. no. 10-1113-01, Mercordia) according to the manufacturer’s protocol. Glycaemic levels were monitored using clinically licensed Contour®Next test strips and the Contour®Next ONE reader (Ascensia Diabetes Care; cat. nos. 84191451 and 85659367, respectively).

### Plots and figures

All plots were prepared in GraphPad Prism 8.4. Figures were designed and assembled using Microsoft PowerPoint and Adobe Illustrator 2022. Data were recorded and managed using Microsoft Excel.

### Statistics and reproducibility

The statistical significance of differences among groups was evaluated with a two-tailed, unpaired Student’s *t*-test using GraphPad Prism 8.4. One-way ANOVA was used to compare differences among more than two groups. Differences are considered statistically significant at *P* < 0.05. The particular statistical test used and the level of significance are reported in the figure legends. All the presented data has been independently repeated five or three times, as mentioned in the figure legends.

### Reporting summary

Further information on research design is available in the Nature Portfolio Reporting Summary linked to this article.

## Supplementary information

Supplementary Information

Description of additional supplementary files

Supplementary Movie 1

Reporting Summary

## Data Availability

The authors declare that all data generated in this study are provided within the paper and in the Supplementary Information/Source Data file. All plasmid information is provided in Supplementary Table 1. The plasmids sequences generated in this study have been deposited in the GenBank database under accession codes “OP966659”, “OP966660”, “OP966661”, “OP966662”, and “OP966663”. Requests for materials should be made to the corresponding author. All plasmids generated in this study are available upon request. Source data are provided with this paper.

## Source data

Source Data
