# Supplementary material for: A versatile bioelectronic interface programmed for hormone sensing
Source: Nat Commun. 2023 May 31;14:3151. doi: 10.1038/s41467-023-39015-1 (PMC10232489; doi:10.1038/s41467-023-39015-1)
Supplement: Supplementary file 1 — Supplementary Information [file 41467_2023_39015_MOESM1_ESM.pdf]

A Versatile Bioelectronic Interface Programmed for Hormone Sensing

Preetam Guha Ray[1], Debasis Maity[1], Jinbo Huang[1], Henryk Zulewski[1,2,3], Martin Fussenegger[1,4]*

[1]ETH Zurich, Department of Biosystems Science and Engineering, Mattenstrasse 26, CH-4058 Basel, Switzerland

[2]Division of Endocrinology, Diabetes and Metabolism, University Hospital Basel, Petersgraben 4, CH–4031 Basel, Switzerland.

[3]Division of Endocrinology and Diabetes, Stadtspital Triemli, Birmensdorferstrasse 497, CH–8063 Zurich, Switzerland.

[4]Faculty of Science, University of Basel, Mattenstrasse 26, CH-4058 Basel, Switzerland.

*Corresponding author. E-mail: fussenegger@bsse.ethz.ch

The PDF file includes:

Supplementary Fig. 1 – 18

Supplementary Table 1 - 2

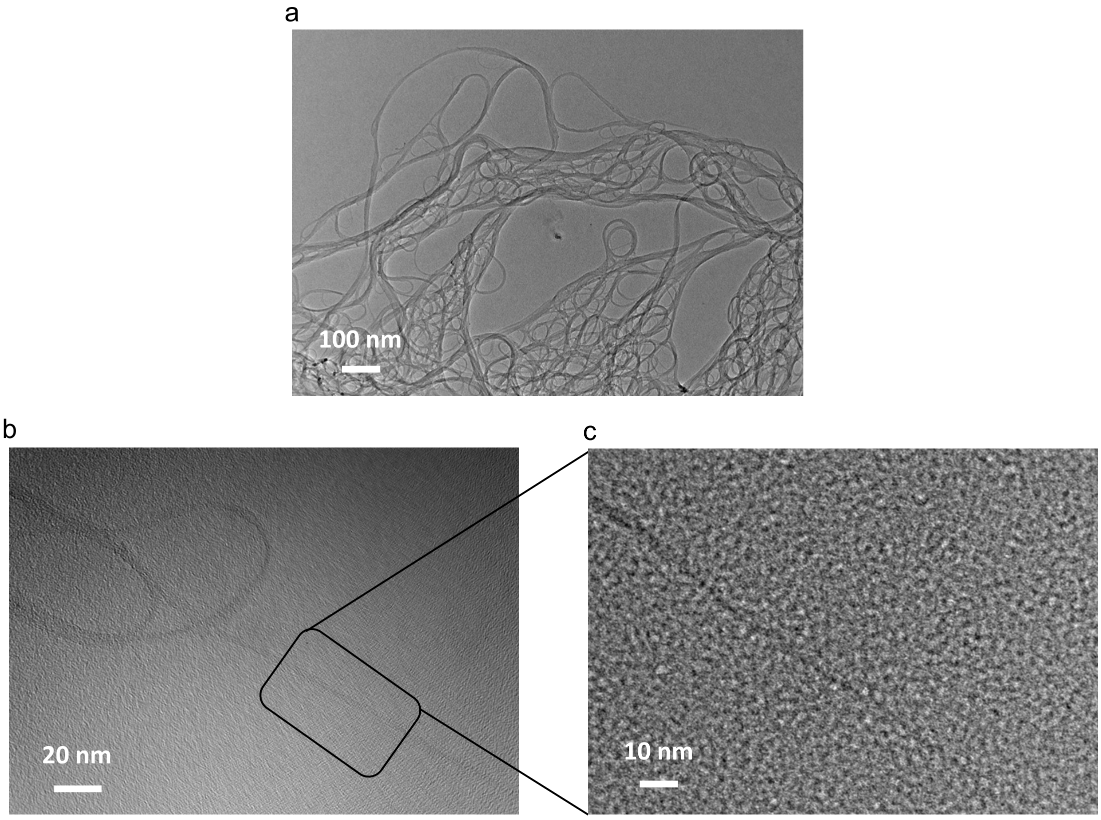

Supplementary Fig. 1 | TEM-based Microstructural Evaluation of SWCNTs. Transmission electron microscopy (TEM) of SWCNTs. **a** Overall distribution of the dispersed carbon nanotubes. **b and c** High-resolution (HR) micrographs showing the single-walled nature and the diameter of the carbon nanotubes. The diameter ranged from 0.8 to 1.20 nm. The region framed by the black lines in **b** is expanded in **c**. The experiments were repeated over 3 independent samples and similar results were obtained from which a representative micrograph is portrayed in the figure.

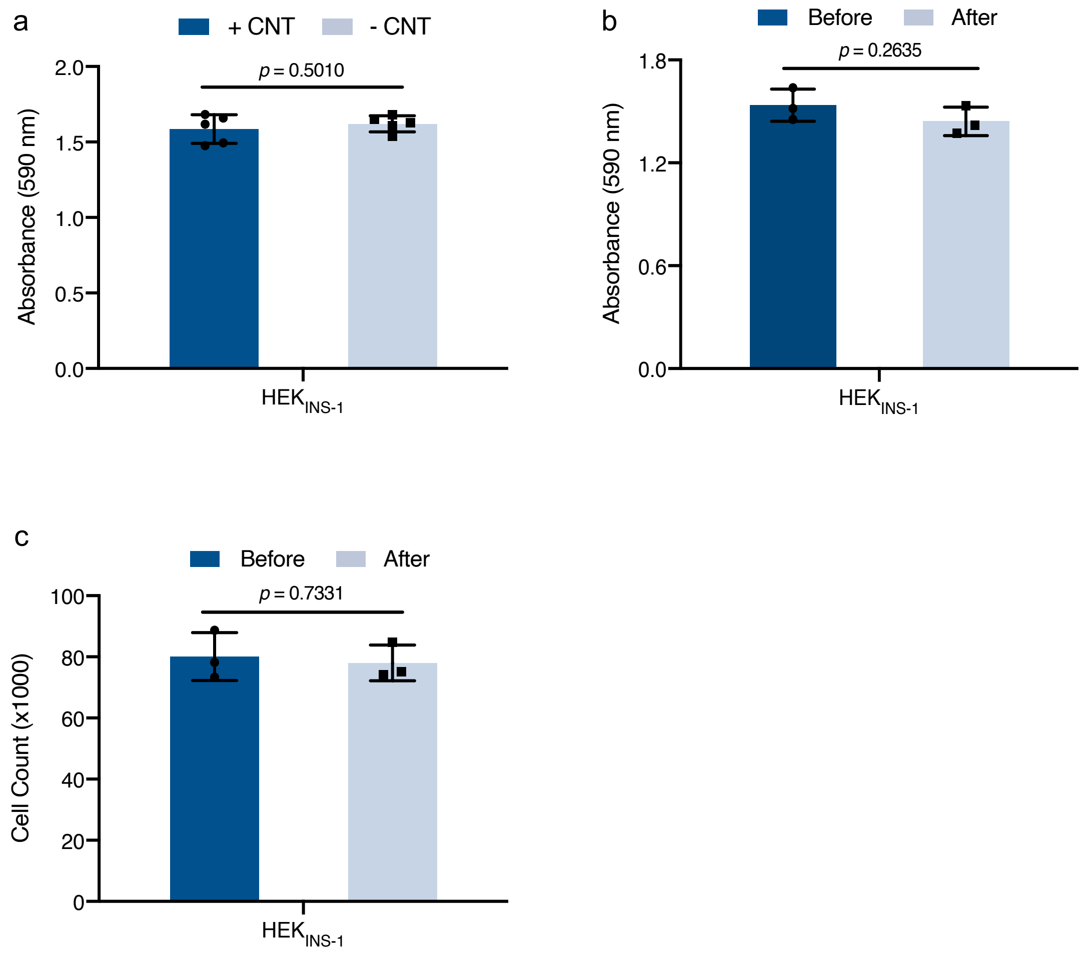

Supplementary Fig. 2| Cell Viability on the VIBE Platform. a Viability of HEK_{INS-1} cells seeded on the working electrode with (+) or without (-) CNT coating was assessed using resazurin assay. Data are presented as mean ± SD of n = 5, biologically independent samples. *p* value was calculated using two-tailed, unpaired Student's *t*-test. **b** Viability was also tested before and after electrochemical measurements. Data are presented as mean ± SD of n = 3, biologically independent samples. *p* value was calculated using two-tailed, unpaired Student's *t*-test. **c** Cell counting before and after the electrochemical analysis was also performed to further support the viability of the HEK_{INS} cells during electrochemical measurement. Data are presented as mean ± SD of n = 3, biologically independent samples. *p* value was calculated using two-tailed, unpaired Student's *t*-test. Source data are provided as a Source Data file.

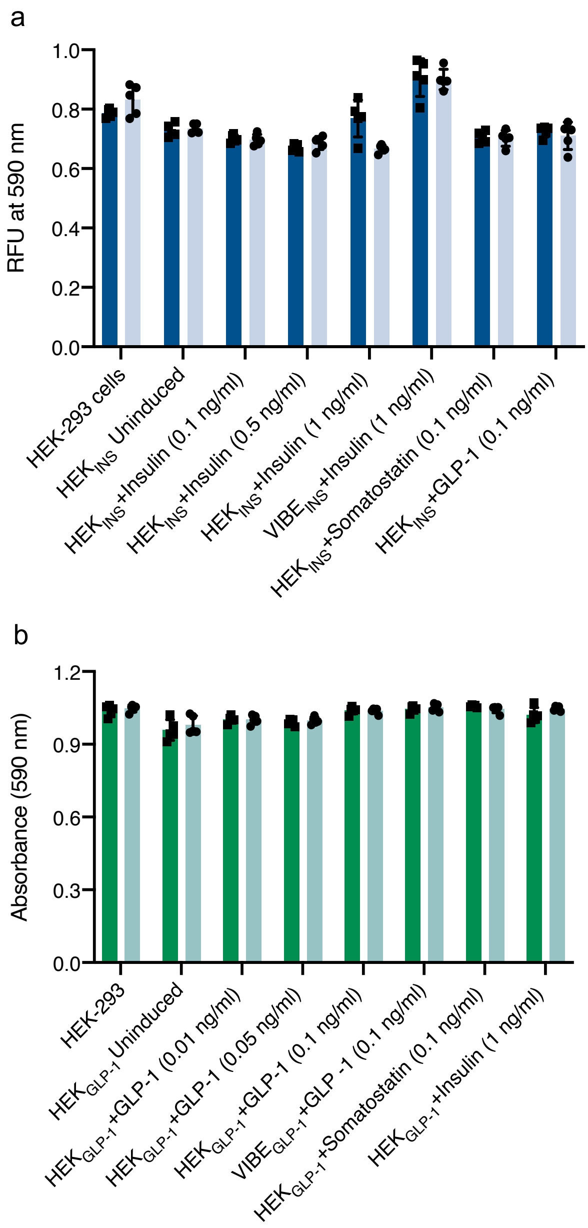

Supplementary Fig. 3 | Cell Viability of HEK$_{INS}$ and HEK$_{GLP\text{-}1}$ cells. a and b Cell viability was assessed by means of resazurin assay for induced, uninduced or non-specifically induced HEK$_{INS}$ and HEK$_{GLP\text{-}1}$ cells in 96-well plate culture. For the insulin-induced system in HEK$_{INS}$ and VIBE$_{INS}$, dark and light blue bars represent uninduced and induced conditions, respectively. For the GLP-1-induced system in HEK$_{GLP\text{-}1}$ and VIBE$_{GLP\text{-}1}$, dark and light green bars represent uninduced and induced conditions, respectively. Data are presented as mean ± SD of n = 5, biologically independent samples. The results demonstrate the cyto-compatibility of the VIBE platform. Source data are provided as a Source Data file.

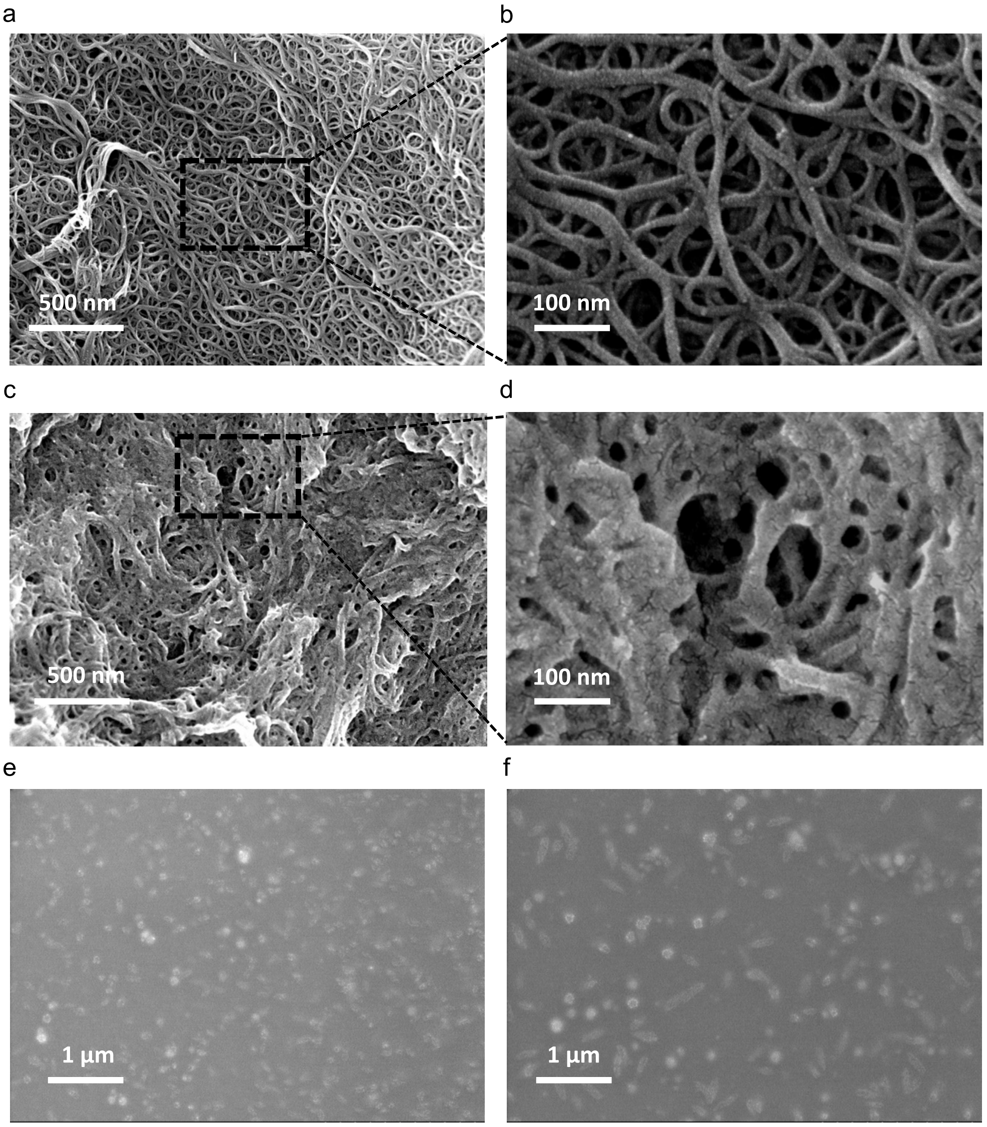

Supplementary Fig. 4 | FESEM-based Microstructural Evaluation of SWCNTs on the VIBE Platform. Micrographs from field effect scanning electron microscopy (FESEM) confirmed SWCNT decoration on the working electrode (WE) (**a and b**) and its retention on WE post trypsinization of HEK_{INS-1} cells from $VIBE_{INS}$ platform (**c and d**). As control experiment, FESEM micrographs of 1x PBS supernatant exposed to SWCNT decorated WE, (**e**) before and (**f**) after centrifuging at 14000 *x g*, clearly depicted absence of SWCNT in the supernatant thus reinstating non-release of SWCNT from the surface of the WE. (b) and (d) are zoomed-in images of the inset from (a) and (c) respectively. (Magnification: (a) and (c) – 50 KX, (b) and (d) – 200 KX, (e) and (f) – 20 KX). Each of the experiments were repeated over 3 independent samples and similar results were obtained from which a representative micrograph is portrayed in the figure.

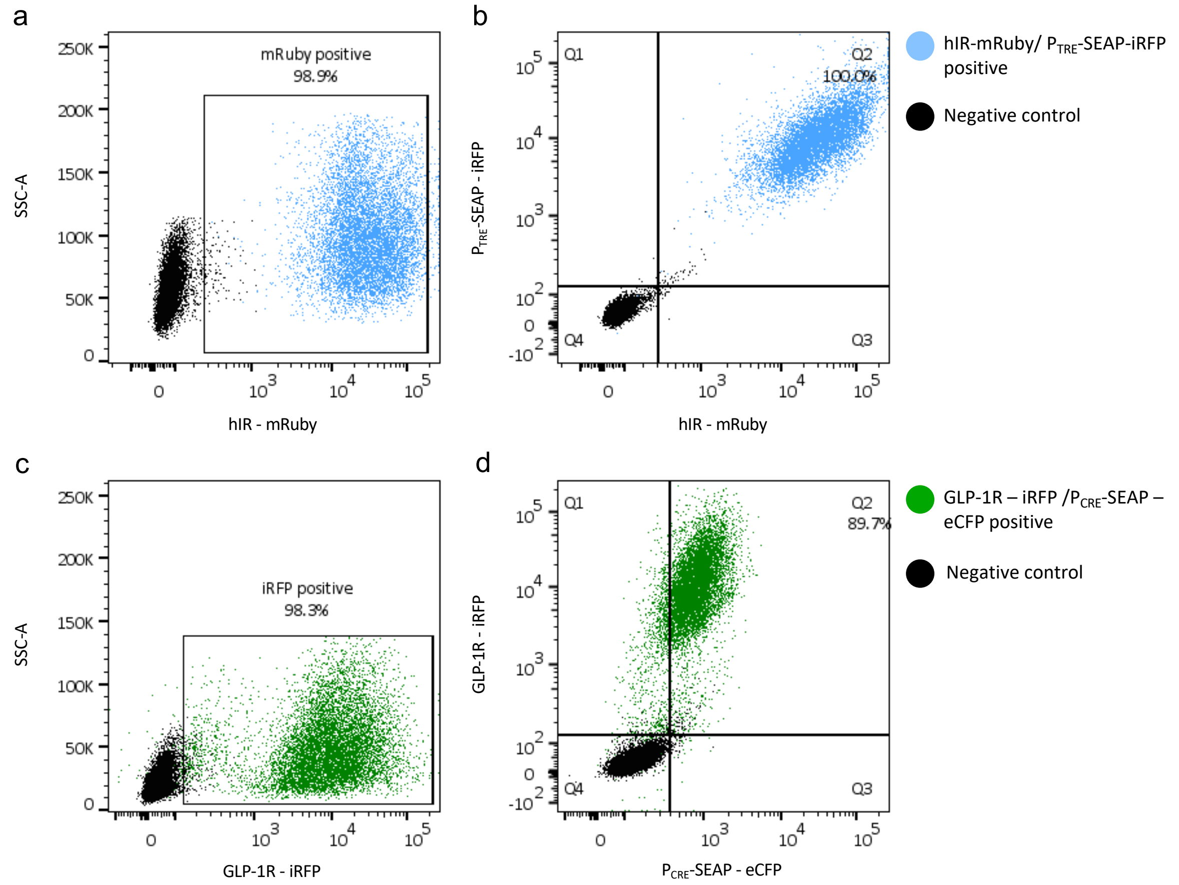

Supplementary Fig. 5 | Fluorescence-activated cell sorting (FACS) analysis. a and c Genomic integration of human insulin receptor (hIR) (ITR-P_{hCMV}-hIR-R-pA:P_{hCMV}-ZeoR-P2A-mRuby-pA-ITR) in $HEK_{INS\text{-}1}$ cells was confirmed using the mRuby positive fluorescent tag (Blue), while genomic integration of GLP-1R (ITR-P_{hCMV}-GLP-1R-pA:P_{hCMV}-BlastR-P2A-iRFP-pA-ITR) in $HEK_{GLP\text{-}1\text{-}1}$ cells was confirmed using the iRFP positive tag (Green). **b** Double-positive $HEK_{INS\text{-}1}$ cells in Q2 stably expressing both the vectors for ITR-P_{hCMV}-hIR-R-pA:P_{hCMV}-ZeoR-P2A-mRuby-pA-ITR and ITR-P_{TRE}-SEAP-pA:P_{hCMV}-BlastR-P2A-iRFP-pA-ITR were selected. **d** Similarly, double-positive $HEK_{GLP\text{-}1\text{-}1}$ cells in Q2 stably expressing both the vectors for ITR-P_{hCMV}-GLP-1R-pA:P_{hCMV}-BlastR-2A-iRFP-pA-ITR and ITR-P_{CRE}-SEAP-pA:P_{RPBSA}-ECFP-P2A-PuroR-pA-ITR were selected. Negative control represents HEK-293 cells without plasmids.

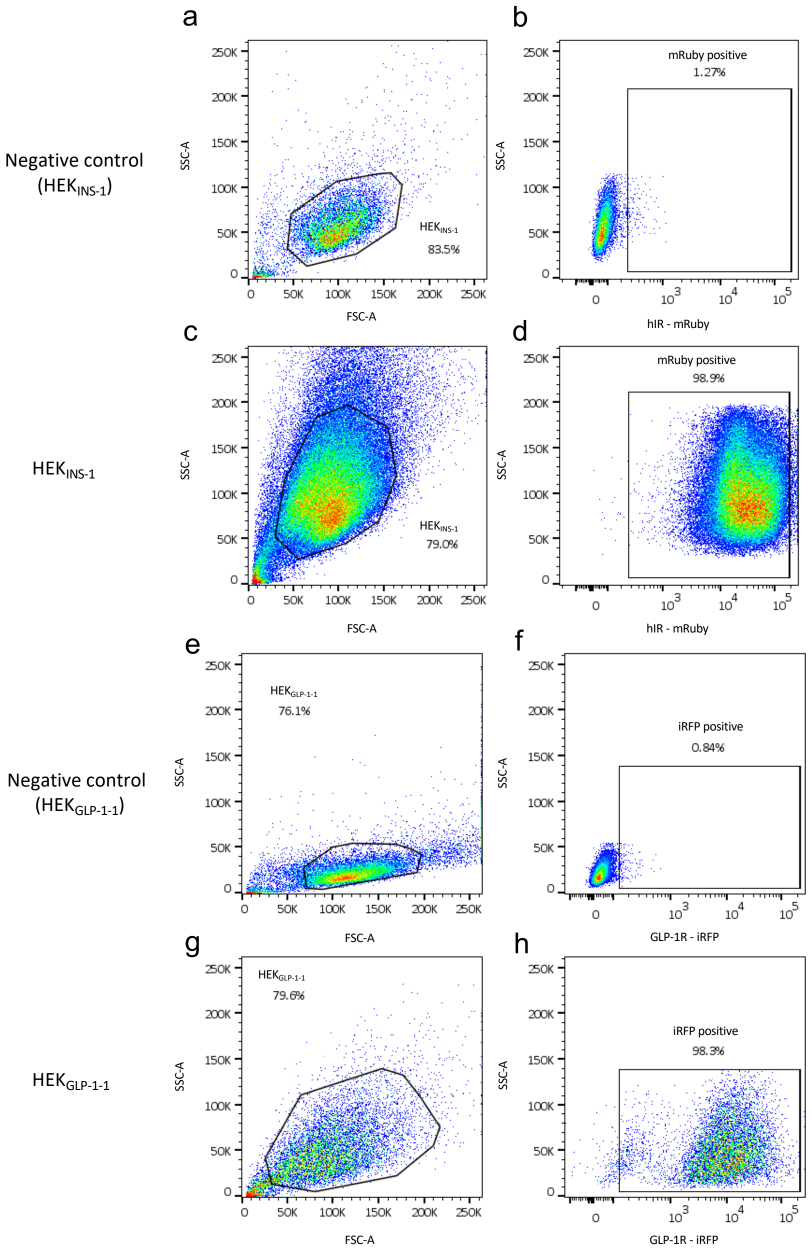

Supplementary Fig. 6| FACS sequential gating strategies. a-d Gating strategy implemented for screening HEK_{INS-1} cells, (hIR-mRuby) while comparing with its negative control (HEK-293T cells without transfection). **e-h** Gating strategy for screening $HEK_{GLP-1-1}$ cells (GLP-1R-iRFP) while comparing with its negative control (HEK-293T cells without transfection).

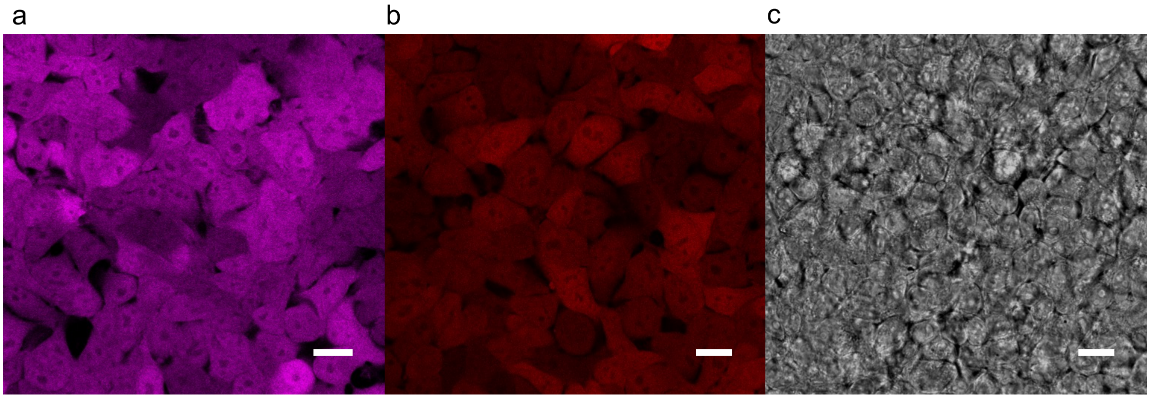

HEK$_{INS-1}$ cells – hIR – mRuby   HEK$_{GLP-1-1}$ cells – GLP-1R – iRFP   Negative control (HEK 293 cells)

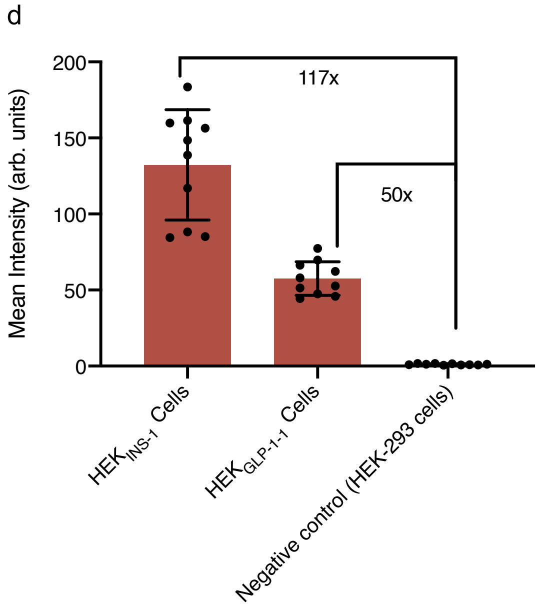

Supplementary Fig. 7 | Image Analysis of HEK$_{INS-1}$ and HEK$_{GLP-1-1}$ cells for transmembrane expression of Receptors. a and b The fluorescence intensity of the mRuby and iRFP tags in the micrographs demonstrate uniform distribution of hIR and GLP-1 receptors, respectively, on the surface of the working electrode (scale bar 10 μm). **c** No emission was observed from native HEK-293 cells (scale bar 10 μm). **d** Image analysis of the acquired fluorescence micrographs (Data are presented as mean ± SD of n = 10) confirmed significant orders of magnitude increase (117x and 50x for insulin and GLP-1 receptors, respectively) of the mean receptor density on the electrode surface when compared with native HEK-293 cells. Source data are provided as a Source Data file. (arb. units - arbitrary units)

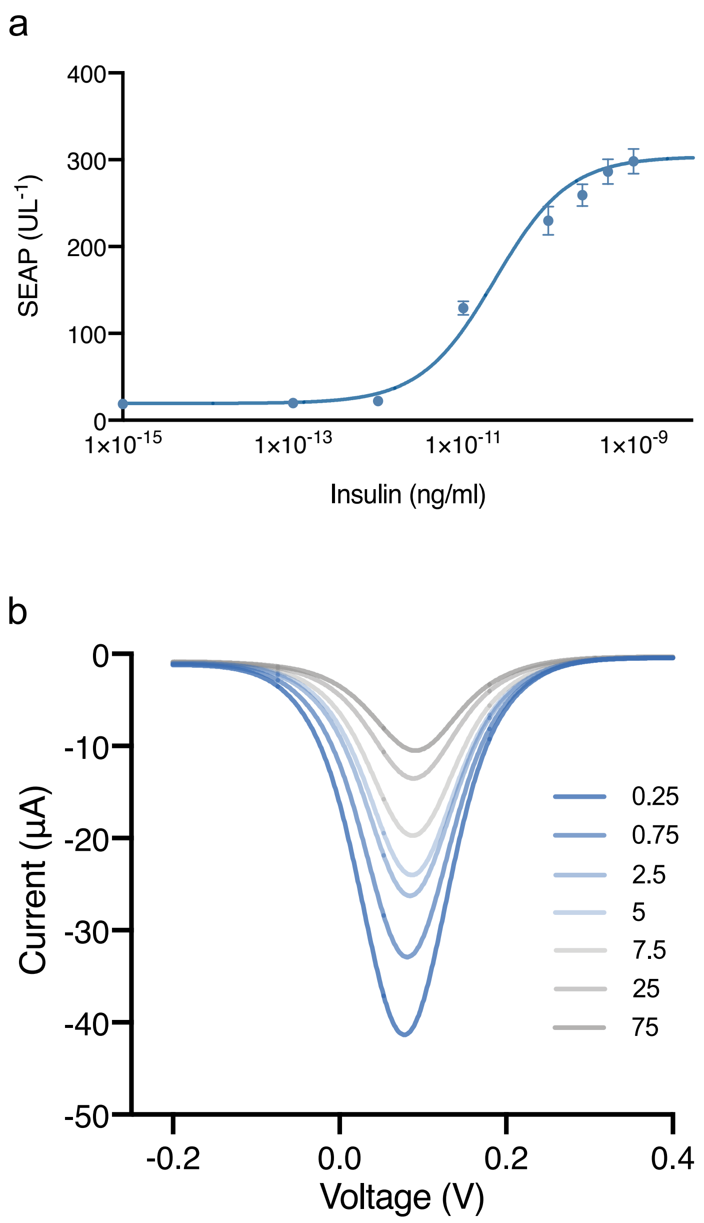

Supplementary Fig. 8 | Dose response of HEK$_{INS}$ cells and DPV analysis of VIBE$_{INS}$. a Dose-response curve for HEK$_{INS}$ cells. Data are presented as mean ± SD of n = 5, biologically independent samples. **b** Differential pulse voltammetry curves for VIBE$_{INS}$ in the presence of the indicated concentrations (ng/ml) of insulin (see also Figure 1d). Source data are provided as a Source Data file.

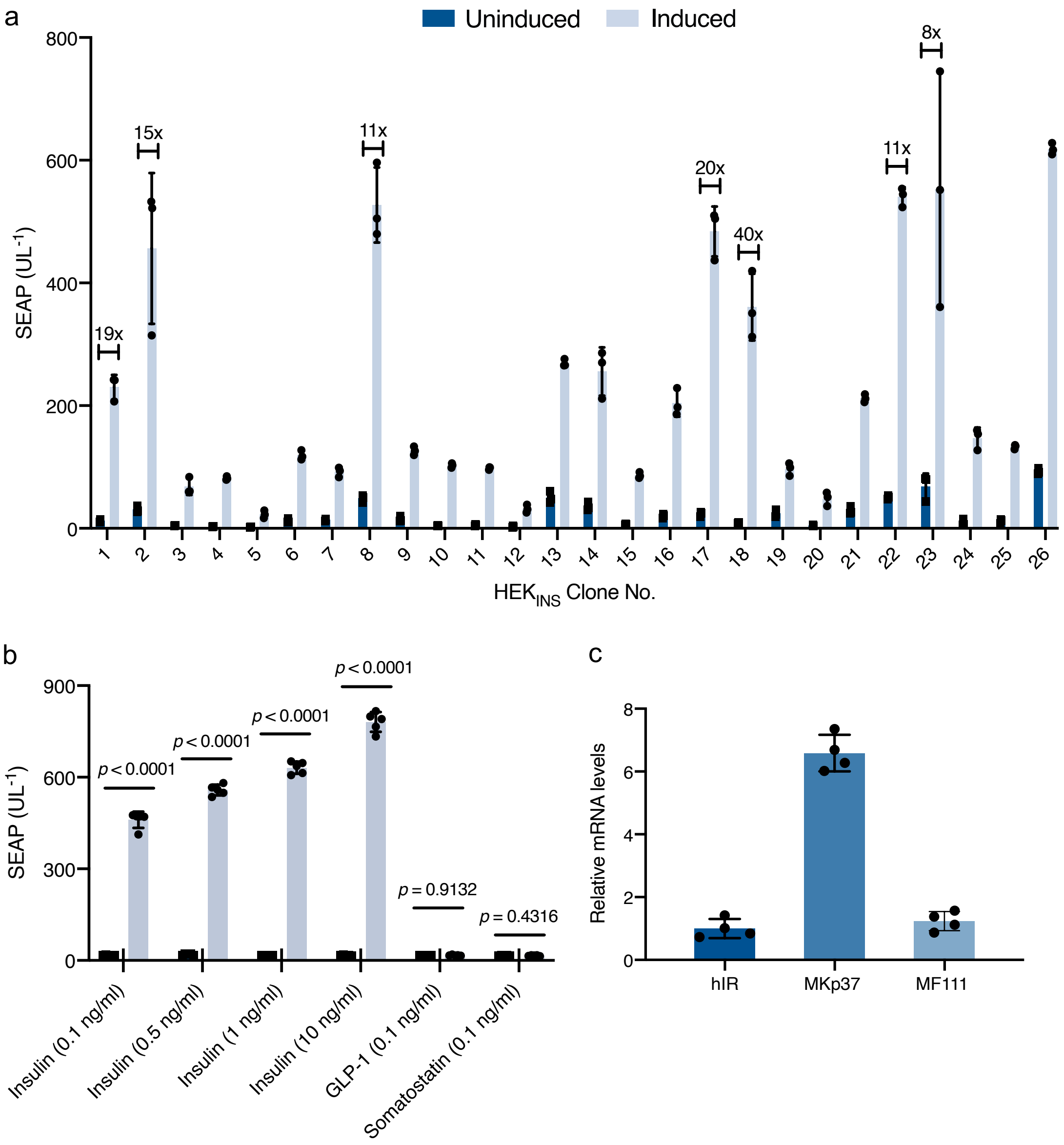

Supplementary Fig. 9 | Stable clone selection for $\text{HEK}_{\text{INS-1}}$ cells. a Clones tested for the VIBE_{INS} interface. HEK-293 cells (5 x 10^4 cells/well) were co-transfected with pJH2026 (ITR-P_{hCMV}-hIR-pA:P_{hCMV}-ZeoR-P2A-mRuby-pA-ITR), pJH2024 (ITR-P_{hCMV}-TetR-ELK1-pA:P_{RPBSA}-ECFP-P2A-PuroR-pA-ITR), and pJH2025 (ITR-P_{TRE}-SEAP-pA:P_{hCMV}-BlastR-P2A-iRFP-pA-ITR) in the molar ratio of 3:1:3 along with 30 ng of Sleeping Beauty transposase expression vector (P_{hCMV}-SB100X-pA). The cells were cultivated in selection medium for 2 weeks, then 26 colonies were picked up at random and grown for 2 passages in selection medium. The colonies were induced with 10 ng/ml insulin, and the SEAP expression was compared with the uninduced basal level. The best-in-class clone no. 18 was selected for further studies. Data are presented as mean ± SD of n = 3, biologically independent samples. **b** Selectivity and specificity of stably

transgenic HEK_{INS} (colony no. 18) were checked by inducing the cells with various concentrations of insulin or somatostatin (0.1 ng/ml) or GLP-1 (0.1 ng/ml). SEAP expression was measured after 24 h. Dark blue bars show basal uninduced expression and light blue bars show induced expression. The results demonstrate high specificity of the system. Data are presented as mean ± SD of n = 5, biologically independent samples. *p* value was calculated using two-tailed, unpaired Student's *t*-test. **c** qPCR results showing the ratio of expression levels among transgenes of *hIR*, MKp37 and MF111 in the $HEK_{INS\text{-}1}$ stable cell line. Data are presented as mean ± SD of n = 4, biologically independent samples. Source data are provided as a Source Data file.

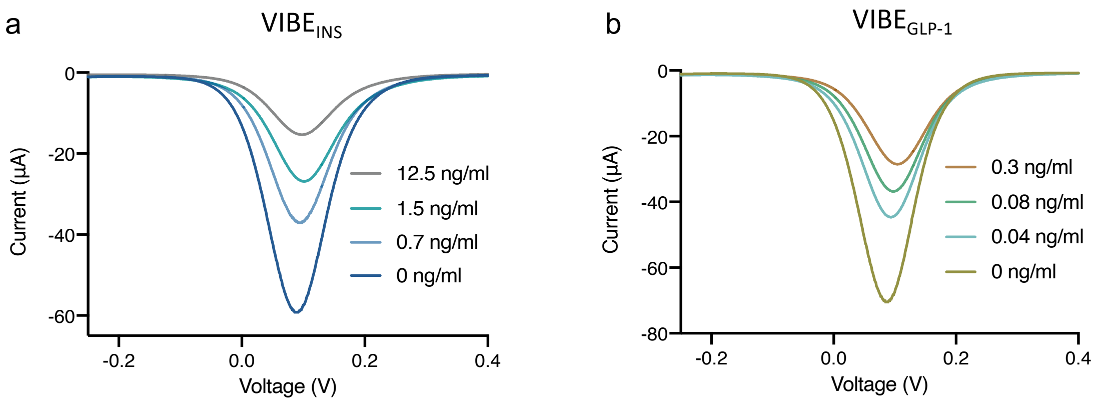

Supplementary Fig. 10 | Evaluation of random concentration on VIBE Platform. a and b The VIBE$_{INS}$ and VIBE$_{GLP-1}$ sensors were tested blind with various concentrations of insulin and GLP-1. The DPV plots show that the output current from the sensors is dependent on the hormone concentration. Source data are provided as a Source Data file.

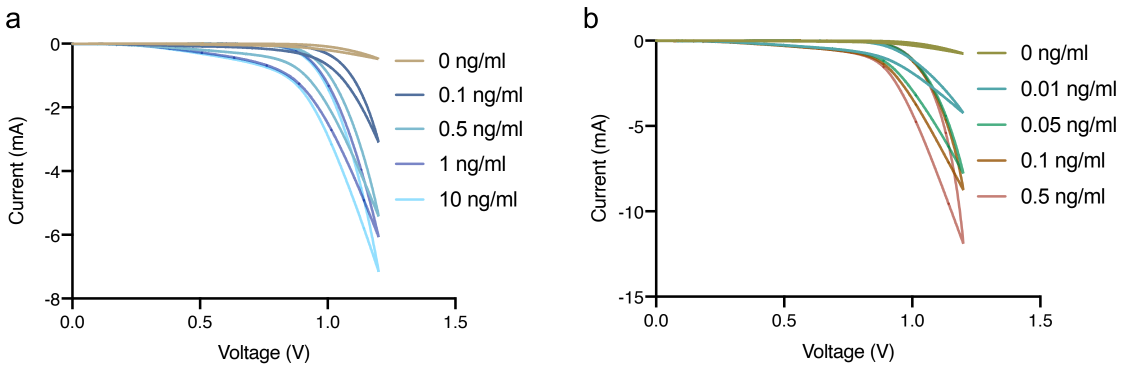

Supplementary Fig. 11 | Electrochemical Analysis of SEAP. a and b Cyclic voltammetry analysis of SEAP levels using the $VIBE_{INS}$ and $VIBE_{GLP\text{-}1}$ platforms after $HEK_{INS\text{-}1}$ and $HEK_{GLP\text{-}1\text{-}1}$ cells seeded on the respective platforms had been induced overnight with various concentrations of insulin or GLP-1, respectively. Source data are provided as a Source Data file.

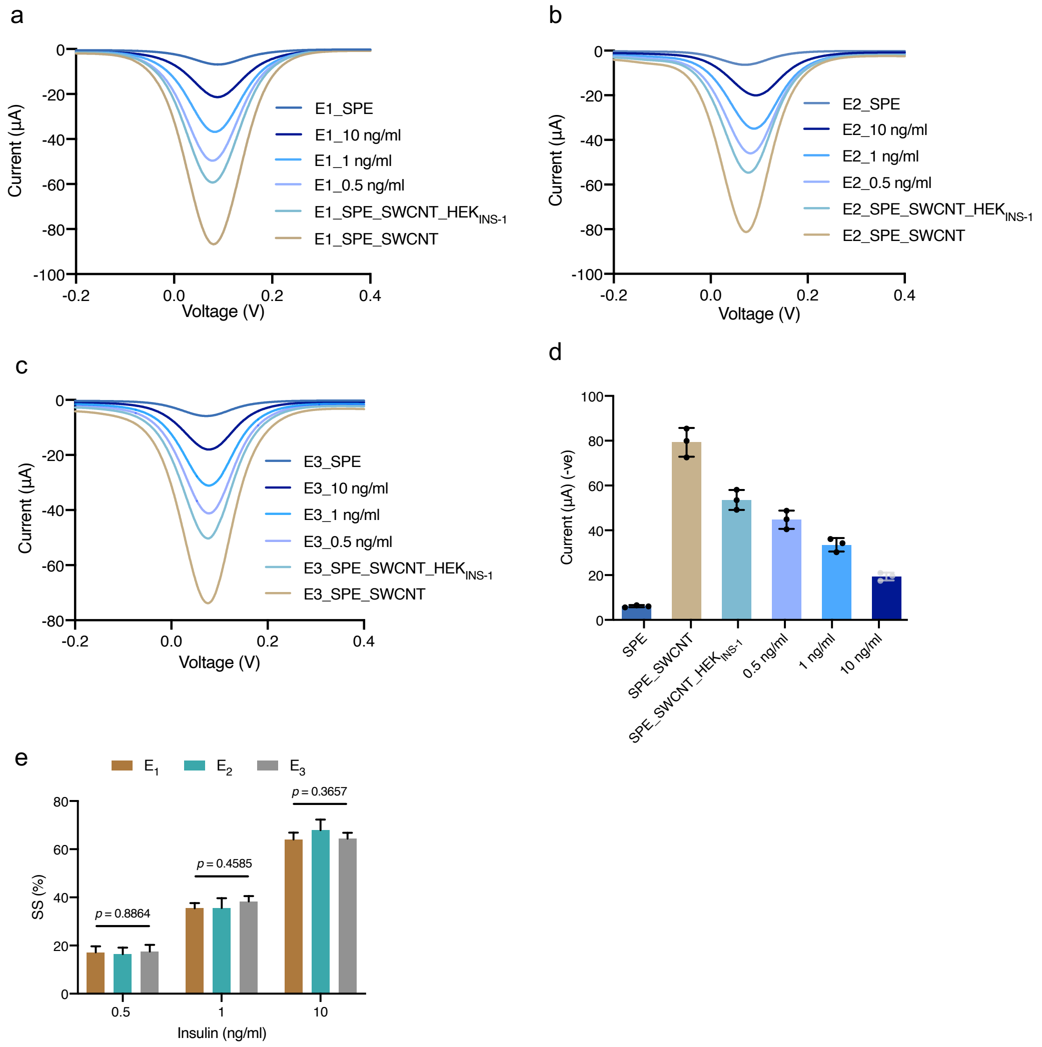

Supplementary Fig. 12 | Repeatability on Multiple Electrodes. a-c DPV plots depicting sensor readings from multiple electrodes, E1, E2 and E3, showing the output current at different stages of sensor fabrication (before and after cell seeding) or when sensing insulin at 0.5, 1 and 10 ng/ml. **d** Plot showing standard deviations of 8-10% in output current from multiple electrodes at different stages of fabrication and sensing. Data are presented as mean ± SD of n = 3, biologically independent samples. **e** Multiple readings on a single electrode were acquired at induced concentrations. no significant difference in signal suppression percentage (SS%) was observed between the 3 electrodes at a given insulin concentration. Data are presented as mean ± SD of n = 3, independent experiments. *p* value was calculated using one-sided ANOVA. Source data are provided as a Source Data file.

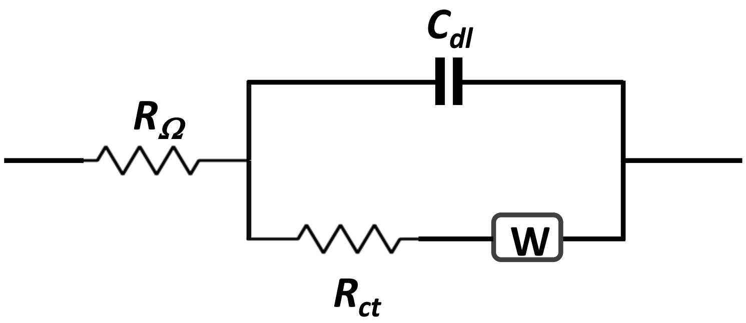

Supplementary Fig. 13 | Equivalent circuit for Nyquist plots obtained for $VIBE_{INS}$ or $VIBE_{GLP-1}$, respectively (Fig. 2G & 3G), used to determine the charge transfer resistance (R_{ct}) for detection of insulin or GLP-1. The equivalent circuit consists of a solution resistance (R_{Ω}), a double-layer capacitance (C_{dl}), a charge transfer resistance (R_{ct}), and the Warburg impedance (Z_W) (W).

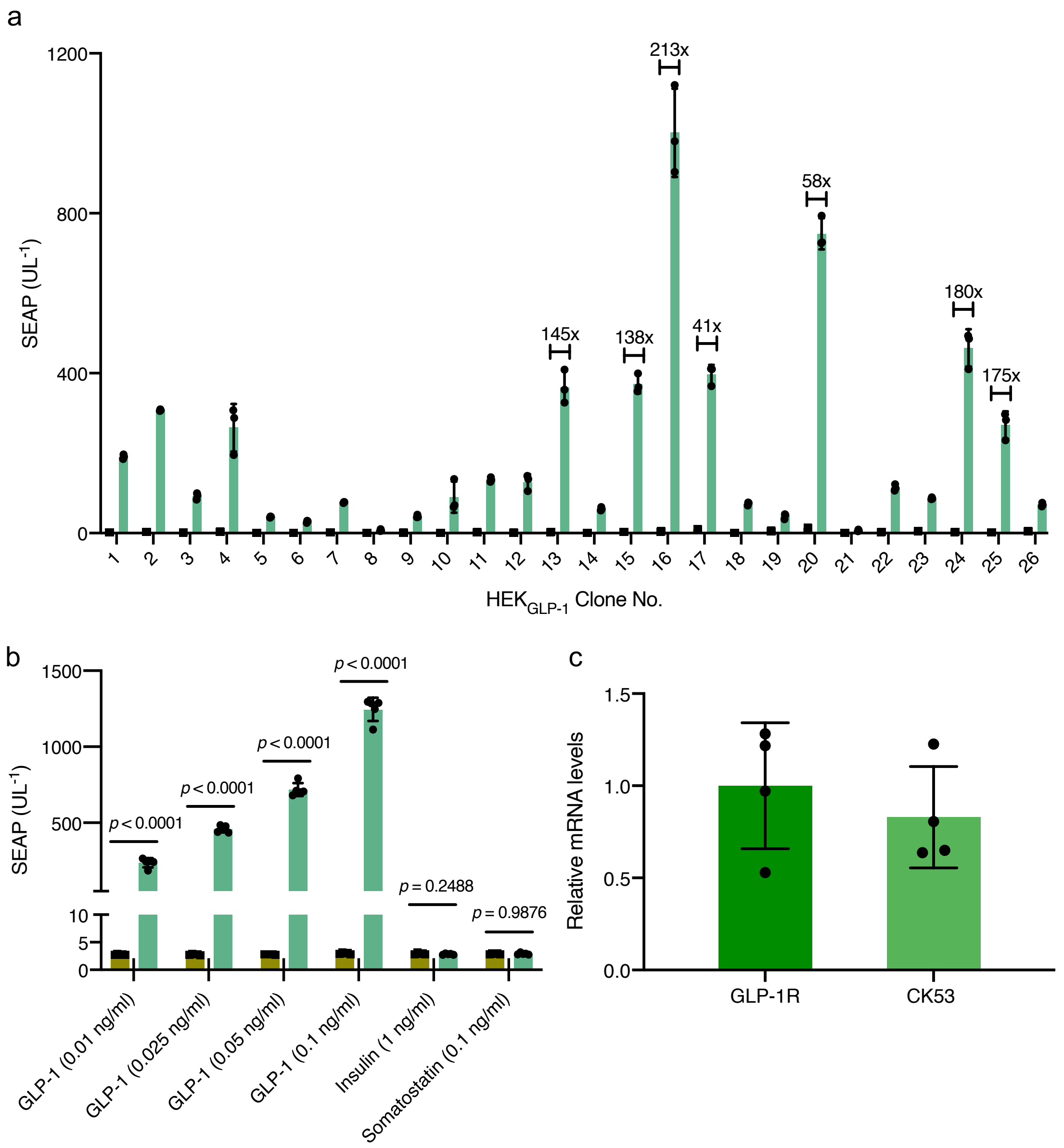

Supplementary Fig. 14 | Stable clone selection for HEK$_{GLP-1-1}$ cells. a For the VIBE$_{GLP-1}$ interface, 5 x 10^4 HEK-293 cells seeded on a 24-well plate were co-transfected with plasmids pJH2023 (ITR-P$_{hCMV}$-GLP-1R-pA:P$_{hCMV}$-BlastR-P2A-iRFP-pA-ITR) and pJH2022 (ITR-P$_{CRE}$-SEAP-pA:P$_{RPBSA}$-eCFP-P2A-PuroR-pA-ITR) in a molar ratio of 1:3 with 30 ng of Sleeping Beauty transposase expression vector (P$_{hCMV}$-SB100X-pA). After culture for 2 weeks in selection medium, 26 colonies were picked up at random and further cultured in selection medium for 2 passages. All the colonies were induced or uninduced and the SEAP expression levels were measured. Data are presented as mean ± SD of n = 3, biologically independent samples. The

best-in-class clone no. 16 was selected for further studies. **b** Selectivity and specificity of clone no. 16 were checked by exposing the cells to various concentrations of GLP-1 or somatostatin (0.1 ng/ml) or insulin (1 ng/ml). SEAP expression was measured after 24 h of induction. Dark green bars show basal uninduced expression and light green bars show induced expression. The results demonstrate high specificity of the system. Data are presented as mean ± SD of n = 5, biologically independent samples. *p* value was calculated using two-tailed, unpaired Student's *t*-test. **c** qPCR results demonstrates that the ratio of expression levels among transgenes of *GLP-1R* and CK53 is 1:0.8 in $HEK_{GLP\text{-}1\text{-}1}$ stable cell line. Data are presented as mean ± SD of n = 4, biologically independent samples. Source data are provided as a Source Data file.

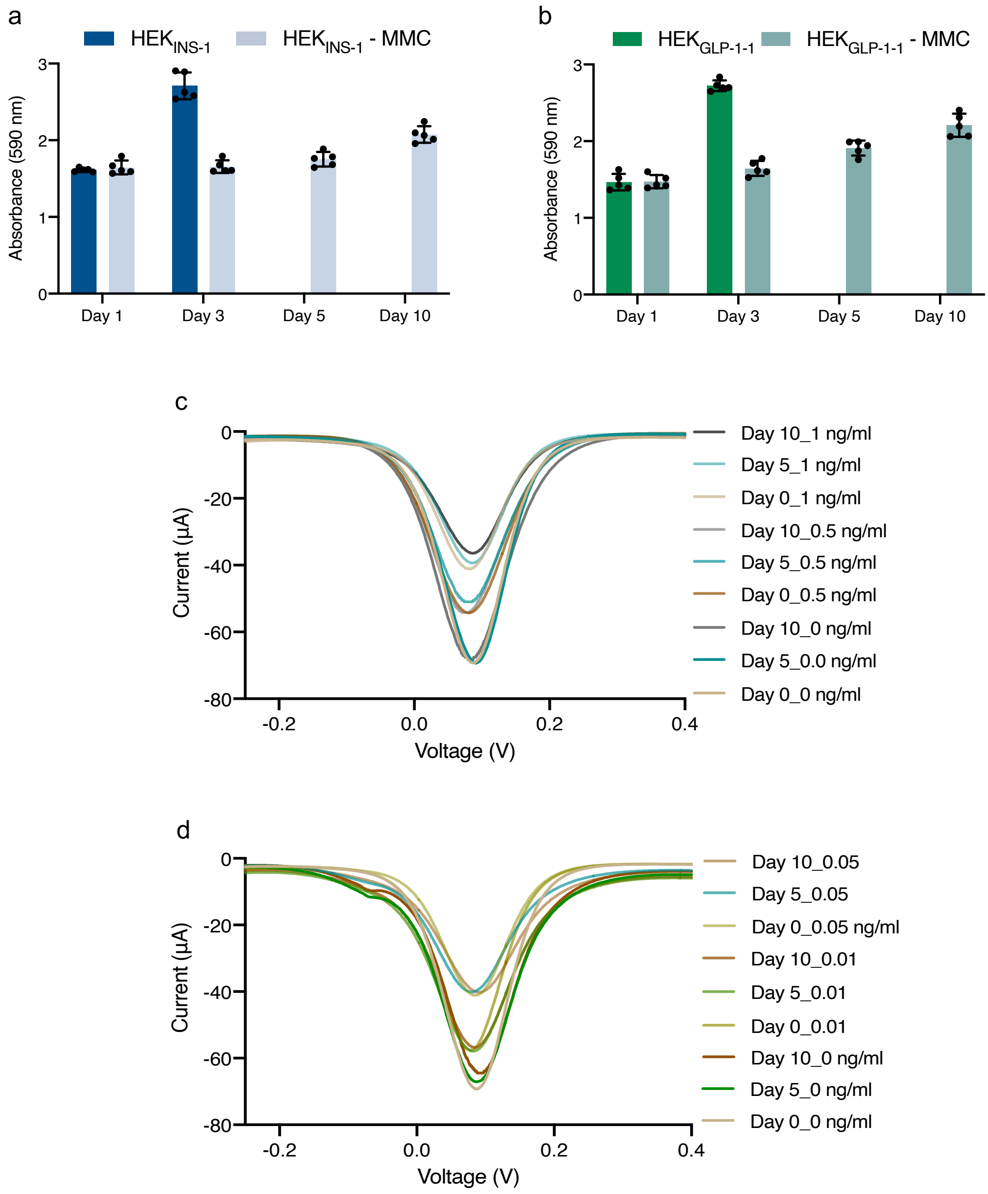

Supplementary Fig. 15 | Storage and Re-usability of VIBE Platform. a and b The viability of mitomycin C (MMC)-treated HEK_{INS-1} and $HEK_{GLP-1-1}$ cells was monitored for 1, 3, 5 and 10 days using resazurin assay. Data are presented as mean ± SD of n = 5, biologically independent samples. **c and d** DPV plots portraying the sensing capability of MMC-treated HEK_{INS-1} and $HEK_{GLP-1-1}$ cells in the $VIBE_{INS}$ and $VIBE_{GLP-1}$ platforms, respectively, at different time points (0, 5, 10 days). Source data are provided as a Source Data file.

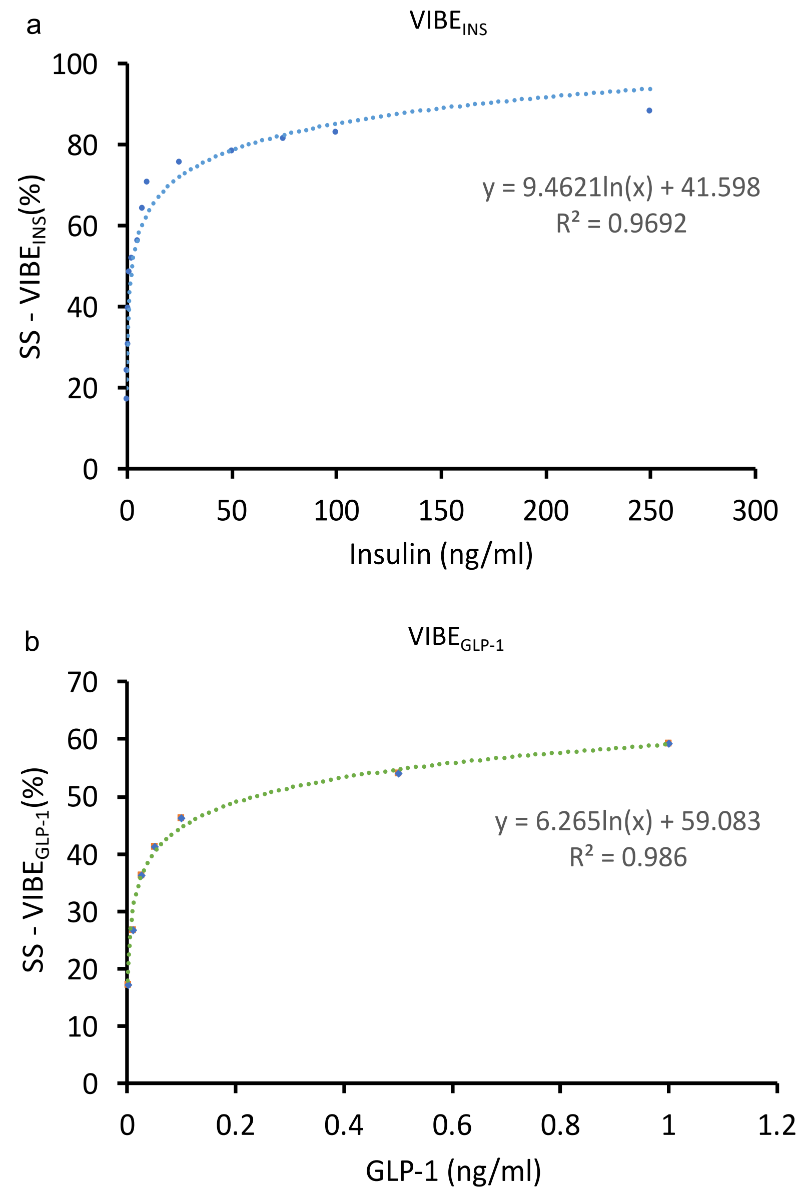

Supplementary Fig. 16 | Calibration Plots. a and b Calibration plots for $VIBE_{INS}$ and $VIBE_{GLP-1}$, used to derive levels of insulin and GLP-1 in mouse blood. SS-$VIBE_{INS}$ (%) and SS-$VIBE_{GLP-1}$ (%) depicts signal suppression (%) for $VIBE_{INS}$ and $VIBE_{GLP-1}$ respectively at varying concentration. Source data are provided as a Source Data file.

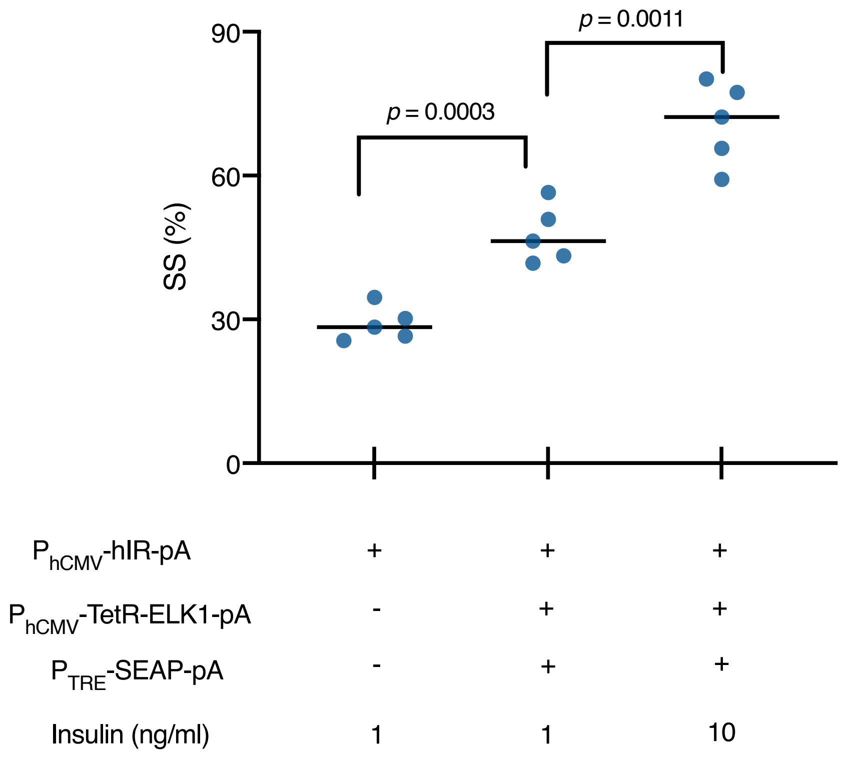

Supplementary Fig. 17 | Efficacy of the complete signaling cascade in engineered HEK_{INS} cells for electrochemical detection of insulin using the $VIBE_{INS}$ platform. In one set of samples, HEK-293 cells transfected with only P_{hCMV}-hIR-pA (insulin receptor) were seeded onto the VIBE platform and used for detection of 1 ng/ml insulin. The results were compared with those obtained with the $VIBE_{INS}$ interface assembled using stably transgenic HEK_{INS-1} cells expressing P_{hCMV}-hIR-pA, P_{hCMV}-TetR-ELK1-pA, P_{TRE}-SEAP-pA plasmids. $VIBE_{INS}$ expressing all the components of the genetic circuit shows a significantly higher signal suppression (SS (%)). Data are presented as mean ± SD of n = 5, biologically independent samples and *p* value was calculated using two-tailed, unpaired Student's *t*-test. Further, the full $VIBE_{INS}$ interface was tested with increasing concentration of the inducer (10 ng/ml) to obtain a higher SS (%). Data are presented as mean ± SD of n = 5, biologically independent samples and *p* value was calculated using two-tailed, unpaired Student's *t*-test. Source data are provided as a Source Data file.

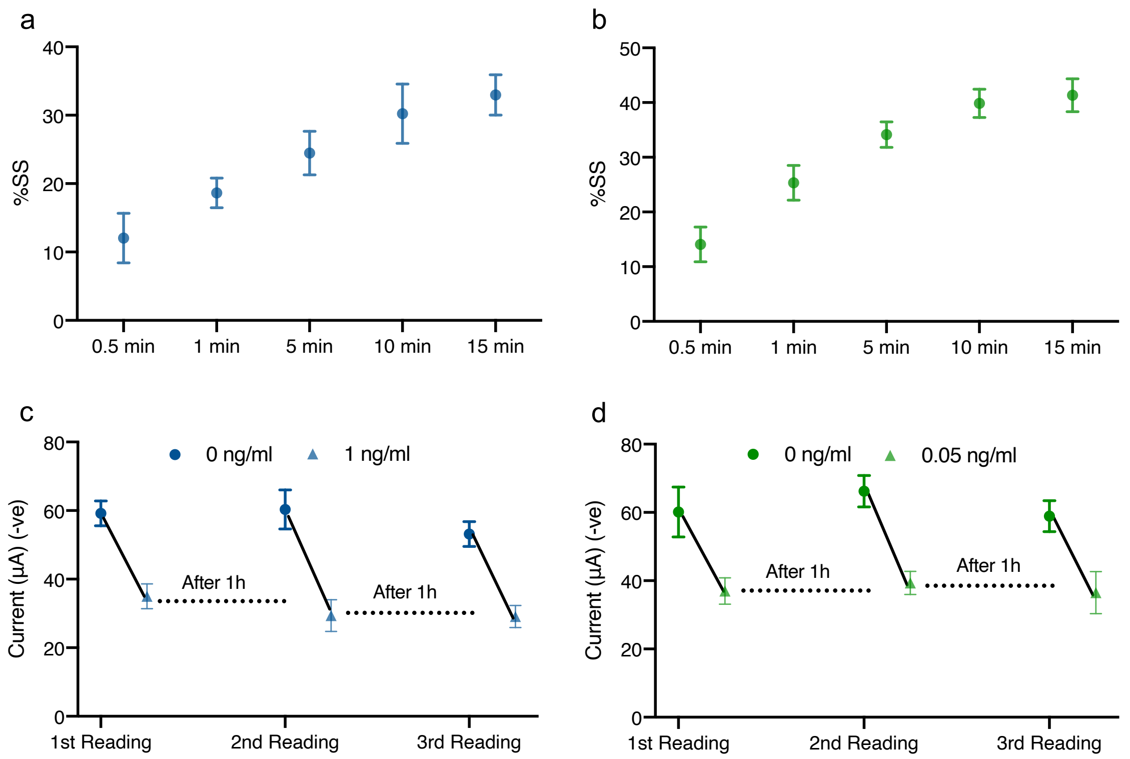

Supplementary Fig. 18 | Response and Recovery of the VIBE platform. a and b Response times of the $VIBE_{INS}$ and $VIBE_{GLP-1}$ platforms where both platforms started to respond within first 0.5 to 1 min and significant increases in %SS values were obtained after 10 min and 5 min, respectively. Data are presented as mean ± SD of n = 3, biologically independent samples. **c and d** $VIBE_{INS}$ and $VIBE_{GLP-1}$ platforms were tested for repeatability of electrochemical sensing using 1 ng/ml and 0.05 ng/ml of insulin and GLP-1, respectively. The repeatability test was performed on the same set of electrodes at intervals of 1 h. Note that the baseline at the uninduced condition (0 ng/ml) shifted back almost to its original position in each case owing to the expression of new receptors to replace the used ones. Data are presented as mean ± SD of n = 3, biologically independent samples. Source data are provided as a Source Data file.

Supplementary Table 1. Plasmids used and designed in this study

Plasmid	Description	Reference
BB6-BlastR	SB100X-specific transposon containing a constitutive BlastR and iRFP expression unit. (ITR-MCS-pA:P_{hCMV}-BlastR-P2A-iRFP-pA-ITR).	Huang et al., unpublished
BB6-PuroR	SB100X-specific transposon containing a constitutive ECFP and PuroR expression unit. (ITR-MCS-pA:P_{RPBSA}-ECFP-P2A-PuroR-pA-ITR).	Huang et al., unpublished
BB6-ZeoR	SB100X-specific transposon containing a constitutive ZeoR and mRuby expression unit. (ITR-MCS-pA:P_{hCMV}-ZeoR-P2A-mRuby-pA-ITR).	Huang et al., unpublished
MKp37	Constitutive mammalian TetR-ELK1 fusion protein expression vector (P_{hCMV}-TetR-ELK1-pA).	Keeley et al. [1]
pcDNA3.1 (+)	Constitutive mammalian expression vector containing a NeoR resistance gene (P_{hCMV}-MCS-pA).	Thermo Fisher Scientific, CA
pCMV-T7-SB100	Constitutive SB100X expression vector (P_{hCMV}-SB100X-pA) (Addgene no. 34879).	Mates et al. [2, 3]
pCK53	CRE-driven SEAP expression vector (P_{CRE}-SEAP-pA).	Kemmer et al. [4]
pGLP-1R	Constitutive mammalian expression vector containing shGLP-1 receptor (shGLP-1R) gene. (P_{hCMV}-shGLP-1R-pA).	Xue et al.[5]
phIR	Constitutive mammalian expression vector containing human insulin receptor (hIR) gene. (P_{hCMV}-hIR-pA).	Ye et al.[6]
pMF111	Mammalian reporter plasmid for TetR-ELK1-induced SEAP expression (P_{TRE}-SEAP-pA).	Fussenegger et al. [7]

pJH42	Constitutive SB100X expression vector in pcDNA3.1(+) backbone. (P_{hCMV}-SB100X-pA).	Huang et al., unpublished
pJH2022	SB100X-specific transposon containing a CRE-driven SEAP expression unit and a constitutive eCFP and PuroR expression unit.<br>(ITR-P_{CRE}-SEAP-pA:P_{RPBSA}-eCFP-P2A-PuroR-pA-ITR).<br>The target fragment was PCR-amplified from pCK53 with OJH2022-GF (5'-TCTAGTCTTAAGAGAT CTACGCGTGCTAGCGCACCAGAC-3') and OJH2022-GR (5'-GCAGGCCGGCCTCAAAGCTTTC ATGTCTGCTCGAAGCGGC-3') and cloned into BB6-PuroR (digested by *Mlu*I/ *Hin*dIII) by Gibson assembly.	This work<br>(GenBank Accession No:<br>OP966659 [https://www.ncbi.nlm.nih.gov/nuccore/OP966659])
pJH2023	SB100X-specific transposon containing a constitutive shGLP-1 receptor expression unit and a constitutive BlastR and iRFP expression unit.<br>(ITR-P_{hCMV}-GLP-1R-pA:P_{hCMV}-BlastR-P2A-iRFP-pA-ITR).<br>The target fragment was PCR-amplified from pGLP-1R with OJH2023-GF (5'-GTCTTAAGAGATC TACGCGTTTGACATTGATTATTGAGTAGTTATTAATAGTAATCAATTA C-3') and OJH2023-GR (5'-GCAGGCCGGCCTCAAAGCTTTCAGCTGCAGGAATTTTGGCAG-3'), and cloned into BB6-BlastR (digested by *Mlu*I/ *Hin*dIII) by Gibson assembly.	This work<br>(GenBank Accession No:<br>OP966660 [https://www.ncbi.nlm.nih.gov/nuccore/OP966660])
pJH2024	SB100X-specific transposon containing a constitutive TetR-ELK1 expression unit and a constitutive eCFP and PuroR expression unit.<br>(ITR-P_{hCMV}-TetR-ELK1-pA:P_{RPBSA}-eCFP-P2A-PuroR-pA-ITR).	This work<br>(GenBank Accession No:<br>OP966661 [https://www.

	The target fragment was PCR-amplified from pCK53 with OJH2023-GF (5'-GTCTTAAGAGATCTACG CGTTTGACATTGATTATTGAGTAGTTATTAATAGTAATCAATTAC-3') and OJH2024-GR (5'-GC AGGCCGGCCTCAAAGCTTGTTACCCGGGACCGGTTCATG-3') and cloned into BB6-PuroR (digested by *Mlu*I/ *Hin*dIII) by Gibson assembly.	ncbi.nlm.nih.gov/nuccore/OP966661])
pJH2025	SB100X-specific transposon containing a O_{TetR7}- and minimal version of P_{hCMV}- driven SEAP expression unit and a constitutive BlastR and iRFP expression unit.<br>(ITR-P_{TRE}-SEAP-pA:P_{hCMV}-BlastR-P2A-iRFP-pA-ITR).<br>The target fragment was PCR-amplified from pMF111 with OJH2025-GF (5'-GTCTTAAGAGATCTAC GCGTCACGAGGCCCTTTCGTCTTCAC-3') and OJH2025-GR (5'-GCAGGCCGGCCTCAAAGCTT TTAACCCGGGTGCGCGG-3') and cloned into BB6-BlastR (digested by *Mlu*I/ *Hin*dIII) by Gibson assembly.	This work<br>(GenBank Accession No:<br>OP966662 [https://www.ncbi.nlm.nih.gov/nuccore/OP966662])
pJH2026	SB100X-specific transposon containing a constitutive human insulin receptor expression unit and a constitutive ZeoR and mRuby expression unit.<br>(ITR-P_{hCMV}-hIR-pA:P_{hCMV}-ZeoR-P2A-mRuby-pA-ITR).<br>The target fragment was PCR-amplified from phIR with OJH2023-GF (5'-GTCTTAAGAGATCTAC GCGTCACGAGGCCCTTTCGTCTTCAC-3') and OJH2026-GR (5'- GCAGGCCGGCCTCAAAGCTT CTAGGAAGGATTGGACCGAGGCAAG-3'), and cloned into BB6-ZeoR (digested by *Mlu*I/ *Hin*dIII) by Gibson assembly.	This work<br>(GenBank Accession No:<br>OP966663 [https://www.ncbi.nlm.nih.gov/nuccore/OP966663])

Abbreviations: **BlastR**, gene conferring blasticidin resistance; **CMV**, cytomegalovirus; **CRE**, cAMP-response element; **CREB1**, CAMP-responsive element binding protein 1; **ECFP**, enhanced cyan fluorescent protein; **EGFP**, enhanced green fluorescent protein; **Elk1**, ETS Like-1 transcription factor; **GLP-1**, glucagon-like peptide 1; **hIR**, modified insulin variant for optimal expression in HEK-293 cells; **iRFP**, near-infrared fluorescent protein; **ITR**, inverted terminal repeats of SB100X; **MCS**, multiple cloning site; **mRuby**, a bright monomeric red fluorescent protein; **NeoR**, neomycin resistance protein; $\mathbf{O_{TetR}}$, TetR-specific operator; **P2A**, picornavirus-derived ribosome skipping sequence optimized for bicistronic expression in mammalian cells; **pA**, polyadenylation signal; **PCR**, polymerase chain reaction; $\mathbf{P_{CRE}}$, CRE-containing synthetic mammalian promoter; $\mathbf{P_{hCMV}}$, human cytomegalovirus immediate early promoter; $\mathbf{P_{hCMVmin}}$, minimal version of P_{hCMV}; $\mathbf{P_{RPBSA}}$: a constitutive synthetic mammalian promotor; $\mathbf{P_{SV40}}$, simian virus 40 promoter; $\mathbf{P_{TRE}}$, O_{TetR}-$P_{hCMVmin}$; **PuroR**, gene conferring puromycin resistance; **SB100X**, optimized Sleeping Beauty transposase; **SEAP**, human placental secreted alkaline phosphatase; **shGLP1**, short human glucagon-like peptide 1; **TetR**, *Escherichia coli* Tn10-derived tetracycline-dependent repressor of the tetracycline resistance gene; **ZeoR**, gene conferring zeocin resistance.

Supplementary Table 2. Primers used for qPCR analysis.

Gene	Forward primer	Reverse primer
GLP-1R	5’-CGAGGGTATCTGGCTGCATA-3’	5’-GCTGACAAGGATGGCTGAAG-3’
SEAP	5’-CCAGACCATTGGCTTGAGTG-3’	5’-TGGTTACCACTCCCACTGAC-3’
hIR	5’-ACCTCGGCCTCATTGAAGAA-3’	5’-GTGGTGAGGTTGTGTTTGCT-3’
TetR-ELK1	5’-AGACTTTCTGCGGAACAACG-3’	5’-GAGAAGCCTTGCTGACACAG-3’
GAPDH	5’-GTCTCCTCTGACTTCAACAGCG-3’	5’-ACCACCCTGTTGCTGTAGCCAA-3’

Supplementary References

1. Keeley, M.B., Busch, J., Singh, R. & Abel, T. TetR hybrid transcription factors report cell signaling and are inhibited by doxycycline. *BioTechniques* **39**, 529-536 (2005).

2. Mátés, L. *et al.* Molecular evolution of a novel hyperactive Sleeping Beauty transposase enables robust stable gene transfer in vertebrates. *Nat. Genet.* **41**, 753-761 (2009).

3. Kowarz, E., Löscher, D. & Marschalek, R. Optimized Sleeping Beauty transposons rapidly generate stable transgenic cell lines. *Biotechnol. J.* **10**, 647-653 (2015).

4. Kemmer, C., Gitzinger, M., Daoud-El Baba, M., Djonov, V., Stelling, J. & Fussenegger, M. Self-sufficient control of urate homeostasis in mice by a synthetic circuit. *Nat. Biotechnol.* **28**, 355-360 (2010).

5. Xue, S*. et al.* A synthetic-biology-inspired therapeutic strategy for targeting and treating hepatogenous diabetes. *Mol. Ther.* **25**, 443-455 (2017).

6. Ye, H. et al. Self-adjusting synthetic gene circuit for correcting insulin resistance. *Nat. Biomed. Eng.* **1**, 005 (2016).

7. Fussenegger, M., Bailey, J.E. & Varner, J. A mathematical model of caspase function in apoptosis. *Nat. Biotechnol.* **18**, 768-774 (2000).
