# Supplementary material for: A versatile bioelectronic interface programmed for hormone sensing
Source: Nat Commun. 2023 May 31;14:3151. doi: 10.1038/s41467-023-39015-1 (PMC10232489; doi:10.1038/s41467-023-39015-1)
Supplement: Supplementary file 2 — Description of additional supplementary files [file 41467_2023_39015_MOESM2_ESM.pdf]

# Description of additional supplementary files

**Supplementary Movie 1 :** Time-lapse fluorescence microscopy video showing on-chip cell viability during an electrochemical experiment. $HEK_{INS-1}$ cells were stained with a live-dead assay kit; live cells were stained with calcein-AM (green fluorescence), while dead cells were stained with ethidium homodimer-1 (ETD-1) (red fluorescence). The presence of live green-fluorescent cells in the left panel and the absence of red-fluorescent dead cells in the right panel confirm the on-chip viability of $HEK_{INS-1}$ cells during the electrochemical measurements. Frames are captured every 5 min up to 30 min. (Scale bar: 10 um).
