# Supplementary material for: A versatile bioelectronic interface programmed for hormone sensing
Source: Nat Commun. 2023 May 31;14:3151. doi: 10.1038/s41467-023-39015-1 (PMC10232489; doi:10.1038/s41467-023-39015-1)
Supplement: Supplementary file 4 — Reporting Summary [file 41467_2023_39015_MOESM4_ESM.pdf]

Corresponding author(s): Prof. Dr. Martin Fussenegger

Last updated by author(s): May 16, 2023

Reporting Summary

Nature Portfolio wishes to improve the reproducibility of the work that we publish. This form provides structure for consistency and transparency in reporting. For further information on Nature Portfolio policies, see our Editorial Policies and the Editorial Policy Checklist.

Statistics

For all statistical analyses, confirm that the following items are present in the figure legend, table legend, main text, or Methods section.

n/a	Confirmed	
☐	☒	The exact sample size (*n*) for each experimental group/condition, given as a discrete number and unit of measurement
☐	☒	A statement on whether measurements were taken from distinct samples or whether the same sample was measured repeatedly
☐	☒	The statistical test(s) used AND whether they are one- or two-sided<br>*Only common tests should be described solely by name; describe more complex techniques in the Methods section.*
☐	☒	A description of all covariates tested
☐	☒	A description of any assumptions or corrections, such as tests of normality and adjustment for multiple comparisons
☐	☒	A full description of the statistical parameters including central tendency (e.g. means) or other basic estimates (e.g. regression coefficient) AND variation (e.g. standard deviation) or associated estimates of uncertainty (e.g. confidence intervals)
☐	☒	For null hypothesis testing, the test statistic (e.g. *F*, *t*, *r*) with confidence intervals, effect sizes, degrees of freedom and *P* value noted<br>*Give P values as exact values whenever suitable.*
☒	☐	For Bayesian analysis, information on the choice of priors and Markov chain Monte Carlo settings
☒	☐	For hierarchical and complex designs, identification of the appropriate level for tests and full reporting of outcomes
☒	☐	Estimates of effect sizes (e.g. Cohen's *d*, Pearson's *r*), indicating how they were calculated

Our web collection on statistics for biologists contains articles on many of the points above.

Software and code

Policy information about availability of computer code

Data collection	Absorbance and luminescence data were collected using Tecan SPARK Reader; Tecan, Männedorf, Switzerland. All electrochemical experiments were carried out using a multichannel CHI 760E electrochemical workstation and data were recorded using chi 760e software. All fluorescent micro-graphs were acquired using a Zeiss LSM 980 fluorescence microscope and the micro-graphs were digitally processed using ZEN Blue 3.5 software. Test of glycaemia were performed using Contour®Next ONE reader. FACs data was acquired using FlowJo software. For image analysis, each image from each ND2 file were segmented into foreground (fluorescence signal) vs. background using the iso data algorithm implementation from scikit-image tool.
Data analysis	All data were analyzed, and plotted using GraphPad Prism 8.4, Microsoft Excel, Microsoft Powerpoint and Adobe Illustrator 2022. FACs data was analyzed using FlowJo 10.5 software. Images were analyzed using scikit-image

For manuscripts utilizing custom algorithms or software that are central to the research but not yet described in published literature, software must be made available to editors and reviewers. We strongly encourage code deposition in a community repository (e.g. GitHub). See the Nature Portfolio guidelines for submitting code & software for further information.

Data

Policy information about availability of data

All manuscripts must include a data availability statement. This statement should provide the following information, where applicable:

- Accession codes, unique identifiers, or web links for publicly available datasets
- A description of any restrictions on data availability
- For clinical datasets or third party data, please ensure that the statement adheres to our policy

The authors declare that all data generated in this study are provided within the paper and in the Supplementary Information/Source Data file. All plasmid information is provided in Supplementary Table S1. The plasmids sequences generated in this study have been deposited in the GenBank database under accession code OP966659, OP966660, OP966661, OP966662, OP966663. Requests for materials should be made to the corresponding author. All plasmids generated in this study are available upon request. Source Data are provided with this paper.

Human research participants

Policy information about studies involving human research participants and Sex and Gender in Research.

Reporting on sex and gender	Blood samples were collected on voluntary basis from members of our research team. 4 male, 1 female took the metabolite challenge test. 5 male took the random blood-insulin level test.
Population characteristics	Blood samples was collected from subjects of varying origins, ages and dietary status.One group of 5 participants (4 male, 1 female, aged between 28 – 35 mean age - 29) took the metabolite challenge test in which blood was collected in the morning before and 1 h after food intake to observe insulin levels under fasting or PP conditions, respectively. Another group of 5 different individuals (5 male, aged between 34 – 60, mean age - 40.2) participated in the random blood-insulin level test.
Recruitment	All volunteers participating in this study were members of our research team. Age group with lower mean age took the metabolite challenge test. Age group with higher mean age took the random blood-insulin level test. The lower mean age group took the metabolite test to facilitate appropriate increase in blood insulin levels thus leading to blood glucose homeostasis at postprandial conditions. Sex or Gender is not a variable that influences human insulin concentrations.
Ethics oversight	All volunteers participating in this study were members of our research team and the collection procedure was performed by a medical practitioner (doctor) and Dr. Henryk Zulewski is also a co-author in the manuscript. The Ethics Committee of Northeastern and Central Switzerland (EKNZ) has classified the use of blood samples by healthy volunteers as a quality assurance project that is not within the scope of the Swiss Federal Act on research involving humans and does therefore not require formal approval by the ethics committee (project-ID: Req-2023-00581 - evaluation of blood insulin and glucose using a bio-electronic interface) based on the stipulations by the Swiss Federal Coordination Office for Human research.

Note that full information on the approval of the study protocol must also be provided in the manuscript.

Field-specific reporting

Please select the one below that is the best fit for your research. If you are not sure, read the appropriate sections before making your selection.

☒ Life sciences ☐ Behavioural & social sciences ☐ Ecological, evolutionary & environmental sciences

For a reference copy of the document with all sections, see nature.com/documents/nr-reporting-summary-flat.pdf

Life sciences study design

All studies must disclose on these points even when the disclosure is negative.

Sample size	No statistical methods were used to predetermine sample size. Sample size was determined based on molecular biology sampling procedure followed in published studies in our field (Scheller L, Nat. Commun. 2020; Krawczyk K, Nat. Commun. 2020; Krawczyk K, Science. 2020). n=5 biologically independent samples were predicted to be sufficient for detecting statistically relevant differences between compared groups in cell culture and animal experiments.
Data exclusions	All data was included.
Replication	Attempts at replication were successful. Replication times are detailed in each figure legend.
Randomization	For cell culture experiments, no covariates based on sample allocations to experimental groups could be observed and no randomization was performed. For each mouse study, animals of the same genetic background were randomly allocated into different experimental groups. For testing VIBE Platform with human blood samples, the lower mean age group took the metabolite test to facilitate appropriate increase in blood insulin levels thus leading to blood glucose homeostasis at postprandial conditions. Age group with higher mean age took the random blood-insulin level test. For the second group covariates didn't play a relevant role since it was a ex-vivo random testing of blood insulin levels with VIBE to compare with the clinically approved standard ELISA procedure.

Blinding	The investigators were not blinded to allocation during experiments and outcome assessment. Blinding was not possible as the same investigator processed the experiments and analyzed the data. Only for one experiment where random concentration of hormone was analyzed. In this in vitro experiment, random concentration of hormone was added blindfold to the sensor by separate individual and concentration was extrapolated from output current of the sensor.

Reporting for specific materials, systems and methods

We require information from authors about some types of materials, experimental systems and methods used in many studies. Here, indicate whether each material, system or method listed is relevant to your study. If you are not sure if a list item applies to your research, read the appropriate section before selecting a response.

Materials & experimental systems

n/a	Involved in the study
☒	☐ Antibodies
☐	☒ Eukaryotic cell lines
☒	☐ Palaeontology and archaeology
☐	☒ Animals and other organisms
☒	☐ Clinical data
☒	☐ Dual use research of concern

Methods

n/a	Involved in the study
☒	☐ ChIP-seq
☐	☒ Flow cytometry
☒	☐ MRI-based neuroimaging

Eukaryotic cell lines

Policy information about cell lines and Sex and Gender in Research

Cell line source(s)	Human embryonic kidney cells (HEK293T, ATCC: CRL-3216).
Authentication	Cell were authenticated by ATCC. All the phenotype of cell lines was frequently checked and controlled by microscopy.
Mycoplasma contamination	HEK293T cells were tested frequently for mycoplasma and confirmed as negative.
Commonly misidentified lines (See ICLAC register)	No commonly misidentified cell lines were used in the study.

Animals and other research organisms

Policy information about studies involving animals; ARRIVE guidelines recommended for reporting animal research, and Sex and Gender in Research

Laboratory animals	BKS-Leprdb/db/JOrlRj (6- to 7-week-old) and C57BL/6JRj (6- to 7-week-old) mice were used in this study and were obtained from Janvier Labs (Saint-Bertheyin, France) and acclimatized for at least 1 week prior to the study.
Wild animals	The study did not involve wild animals.
Reporting on sex	BKS-Leprdb/db/JOrlRj and C57BL/6JRj male mice were used in this study. The selection of mice was based on sampling procedures and studies followed in published studies in our field in order to keep similar metabolic conditions (Scheller L, Nat. Commun. 2020; Krawczyk K, Nat. Commun. 2020; Krawczyk K, Science. 2020).
Field-collected samples	The study did not involve field-collected samples.
Ethics oversight	All animal experiments were performed in accordance with the Swiss animal welfare legislation and approved by the Veterinary Office of the Canton Basel-Stadt, Switzerland (license number: 2996_34477) and conducted by P.G.R (license number: LTK 5507) at the Department of Biosystems Science and Engineering (D-BSSE) of the ETH Zurich in Basel, Switzerland.

Note that full information on the approval of the study protocol must also be provided in the manuscript.

Flow Cytometry

Plots

Confirm that:

☒ The axis labels state the marker and fluorochrome used (e.g. CD4-FITC).

☒ The axis scales are clearly visible. Include numbers along axes only for bottom left plot of group (a 'group' is an analysis of identical markers).

☒ All plots are contour plots with outliers or pseudocolor plots.

☒ A numerical value for number of cells or percentage (with statistics) is provided.

Methodology

Sample preparation	The sample preparation is described in details in the Methods section of the manuscript. Briefly, a polyclonal population of HEK293T cells were prepared in FACS sorting buffer (PBS with 0.2%FBS) and were filtrated before sorting via a 5 ml Polystyrene tube with Cell-Strainer Cap 35µm, Falcon #352235. Cells were kept on ice and were sorted into two different subpopulations according to their mRuby and iRFP or iRFP and eCFP fluorescence intensities. Cells expressing both fluorophores simultaneously were plotted in Q2 and were sorted in a 6-well plate containing collection media (DMEM + 10% FBS+ 500 ug/ml Pen-Strep). The cells were regularly checked for contamination.
Instrument	Flow cytometry analysis was performed on a FACSAria Fusion Cell Sorter, Becton Dickinson, New Jersey, USA
Software	Flow cytometry data were analyzed with FlowJo 10.5 software.
Cell population abundance	Using fluorescent output, positive cells for mRuby and iRFP or iRFP and eCFP signals were sorted into 6-well plate containing collection media (DMEM + 10% FBS+ 500 ug/ml Pen-Strep) as described in sample preparation. 50-60% of sorted cells could grow and make monolayer in a well of 6-well plate. These monoclonal cell populations were screened for Insulin and GLP-1 based on the SEAP production. The results are included in supplementary file for more clarity
Gating strategy	A comprehensive report is attached in supplementary file. Gating for positive cells was performed based on wild-type cells expressing no fluorophore.

☒ Tick this box to confirm that a figure exemplifying the gating strategy is provided in the Supplementary Information.

March 2021
